# Wireless charging-mediated angiogenesis and nerve repair by adaptable microporous hydrogels from conductive building blocks

**DOI:** 10.1038/s41467-022-32912-x

**Published:** 2022-09-02

**Authors:** Ru-Siou Hsu, Ssu-Ju Li, Jen-Hung Fang, I-Chi Lee, Li-An Chu, Yu-Chun Lo, Yu-Jen Lu, You-Yin Chen, Shang-Hsiu Hu

**Affiliations:** 1 Department of Biomedical Engineering and Environmental Sciences, National Tsing Hua University, Hsinchu, 300044 Taiwan; 2 Department of Biomedical Engineering, National Yang Ming Chiao Tung University, Taipei, 112304 Taiwan; 3 Brain Research Center, National Tsing Hua University, Hsinchu, 300044 Taiwan; 4 The Ph.D. Program for Neural Regenerative Medicine, College of Medical Science and Technology, Taipei Medical University, Taipei, 11031 Taiwan; 5 Department of Neurosurgery, Chang Gung Memorial Hospital, College of Medicine Chang Gung University, Taoyuan, 33305 Taiwan; 6 College of Medicine, Chang Gung University, Kwei-San, Taoyuan, 33302 Taiwan; 7 Medical Device Innovation and Translation Center, National Yang Ming Chiao Tung University, Taipei, 112304 Taiwan

**Keywords:** Developmental neurogenesis, Biomedical engineering, Regeneration and repair in the nervous system, Biomedical materials, Brain injuries

## Abstract

Traumatic brain injury causes inflammation and glial scarring that impede brain tissue repair, so stimulating angiogenesis and recovery of brain function remain challenging. Here we present an adaptable conductive microporous hydrogel consisting of gold nanoyarn balls-coated injectable building blocks possessing interconnected pores to improve angiogenesis and recovery of brain function in traumatic brain injury. We show that following minimally invasive implantation, the adaptable hydrogel is able to fill defects with complex shapes and regulate the traumatic brain injury environment in a mouse model. We find that placement of this injectable hydrogel at peri-trauma regions enhances mature brain-derived neurotrophic factor by 180% and improves angiogenesis by 250% in vivo within 2 weeks after electromagnetized stimulation, and that these effects facilitate neuron survival and motor function recovery by 50%. We use blood oxygenation level-dependent functional neuroimaging to reveal the successful restoration of functional brain connectivity in the corticostriatal and corticolimbic circuits.

## Abstract (web summary)

Traumatic brain injury can cause long-term disability and thus constitutes a substantial healthcare burden worldwide. Here, the authors report a conductive microporous hydrogel to improve angiogenesis and recovery of brain function in traumatic brain lesions.

## Introduction

Traumatic brain injury (TBI) is the most common cause of severe long-term disability in industrial countries^1^. Approximately 50 million people suffer from TBI, and TBI costs up to $400 billion worldwide due to its consequences of chronic dysfunctions of mood and behavior^2^. The critical reason for these consequences is that there is still a considerable barrier to tissue regeneration following injury to the central nervous system. Most patients with TBI experience persistent injury-related difficulties in daily life one year after injury, and these difficulties commonly accompanied by impairments in physical, cognitive, and psychosocial functions^3^. In the TBI cavity, there is no physical matrix support for cell penetration into the trauma and tissue regeneration^4^. Furthermore, large numbers of infiltrating microglia, macrophages and activated astrocytes increase inflammation, leading to glial scarring and neuronal death in the peri-trauma area^5–7^. These astrocytic and fibrotic scars may impede recovery in the TBI cavity and result in cerebral atrophy (brain shrinkage) in the motor/sensory cortex. Additionally, it is difficult to establish a long-lasting repair response that includes angiogenesis and neurogenesis in the damaged tissue in the brain. To date, the clinical treatment of in TBI still lacks an effective medical therapy for cerebral atrophy and the promotion of long-term recovery^8^.

Following brain injury, inflammation increases the production of reactive oxygen species (ROS), which are associated with severe cerebral ischemia and worsening of symptoms such as hypoxia, hypotension, and hematomas^9^. A potential strategy to address these issues is to engineer an injectable hydrogel that dramatically eliminates inflammation and induces angiogenesis/neurogenesis at different stages of tissue regeneration. In this regard, hyaluronic acid-based hydrogels loaded with an antioxidant provided unique features to reduce oxidative stress and induce neuronal migration at the injury site^10^. However, traditional nonporous hydrogels do not appropriately mimic the mechanical properties of native tissues that are necessary for orchestrating cells, which might cause insufficient tissue support and promote fibrosis^11–13^. To overcome this limitation, injectable beads capable of spontaneously forming interconnected pores and tuning their physicochemical properties were developed^14, 15^. For example, Segura et al. reported that annealed microporous gels accelerate cell penetration and nerve repair processes by reducing gliosis and enhancing vascularization in the stroke cavity^16, 17^. In our previous work, an adaptable microporous hydrogel that released gradients of neuron growth factors in nerve conduits also successfully promoted peripheral nerve axon outgrowth of up to 4.7 mm within one week^18^. Despite the recent interest in porous gel engineering, these gels still only minimally promote angiogenesis/neurogenesis in complex TBI repair, which limits long-term functional recovery^19^.

Electrical deep brain stimulation (DBS) has been approved by Food and Drug Administration (FDA) for treating intractable brain disorders by directly controlling brain circuit dynamics^20–22^. Electric currents offering on-demand biocues can trigger different biological processes and programming^23^. Recently, a noninvasive current was generated by an external high-frequency magnetic field (HFMF), which was used to charge nanoconductors, such as gold nanoparticles or graphene oxide sheets, to promote lineage reprogramming of dopamine neurons^24, 25^. Under an HFMF, the Lorentz force acts on the moving charge carrier and causes periodic lattice vibrations due to the oscillating eddy currents. Such induced electric currents can control drug release or promote osteogenesis and neurogenesis^26^. Since the currents are mainly concentrated near the surface area of conductors and exponentially decrease in the depth direction^27^, a nanoconductor with effectively inducing current is needed. Furthermore, electroactuated particles, such as gold, silver nanoparticles, carbon nanotubes and graphene are typically used as additives to modify hydrogels to impart conductivity. However, most nanoparticles embedded in gels cannot transport electrons to cells, and they also affect the gelation ability and alter the mechanical properties of hydrogels.

**Fig. 1:**
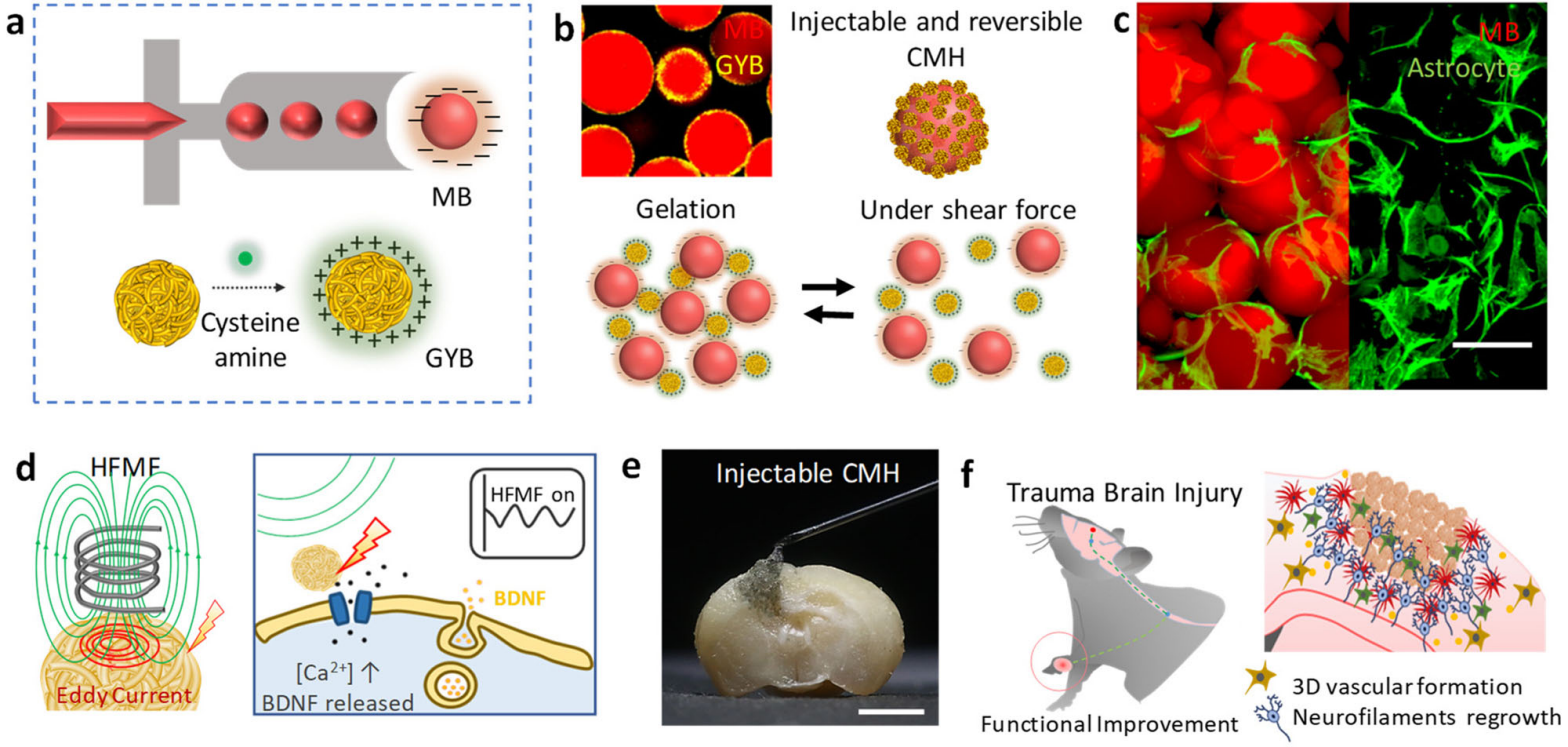
Formation of an adaptable conductive microporous hydrogel (CMH). **a** Schematic illustration of monodispersed gelatin methacrylamide (GelMA) injectable building blocks (microbeads, MBs) formation (upper); the process of synthesizing cys-GYB (lower). **b** Schematic illustration of injectable and reversible CMH. **c** Fluorescence image of a cell-laden micronetwork scaffold. Scale bar: 100 μm. **d** Schematic illustration of eddy current induced by HFMF (left) and promote BDNF releasing (right). **e** A photograph image represents the characteristic of injectable CMH into irregular cavity. Scale bar: 1.5 mm. **f** Schematic illustration of CMH with an electrical stimulation and axonal neurofiliments regrow in vivo.

Here, the electromagnetized gold nanoyarn balls (GYBs)-coated injectable building blocks (microbeads, MBs) were developed that combine the features of surface roughness (a component of surface texture) and remote electrical stimulation to promote angiogenesis and neurogenesis at the damaged tissue during different stages of brain tissue regeneration. The roughness surface upon an electromagnetized stimulus could induce the effective current known as the skin effect owing to the induced eddy current exponential decreasing with surface depth^36, 37^. This conductive microporous hydrogel (CMH), which is composed of positively charged GYBs (electromagnetized generators) and negatively charged microbeads (MBs, interconnected pore formation and pore size control in the brain cavity), can be reshaped and reassembled by shear-thinning force, facilitating the construction of a three-dimensional porous scaffold (Fig. 1a–b). Via the charge interactions, the GYBs were exposed on the surfaces of MBs to contact the cells directly. The interconnected CMH possesses suitable micropores for prompting cell migration and mechanical support for cell penetration (Fig. 1c). Upon application of an HFMF, the induced eddy currents generated by the GYBs on gel guide to produce brain-derived neurotrophic factor (BDNF) and calcium ion permeability to enhance neuroprotective processes (Fig. 1d)^28–30^. The synergistic effects of electrical stimulation and cell penetration in this injectable CMH in the brain cavity can improve angiogenesis, neurogenesis, and functional recovery in vivo (Fig. 1e–f).

## Results

### Synthesis and characterization of CMH

**Fig. 2:**
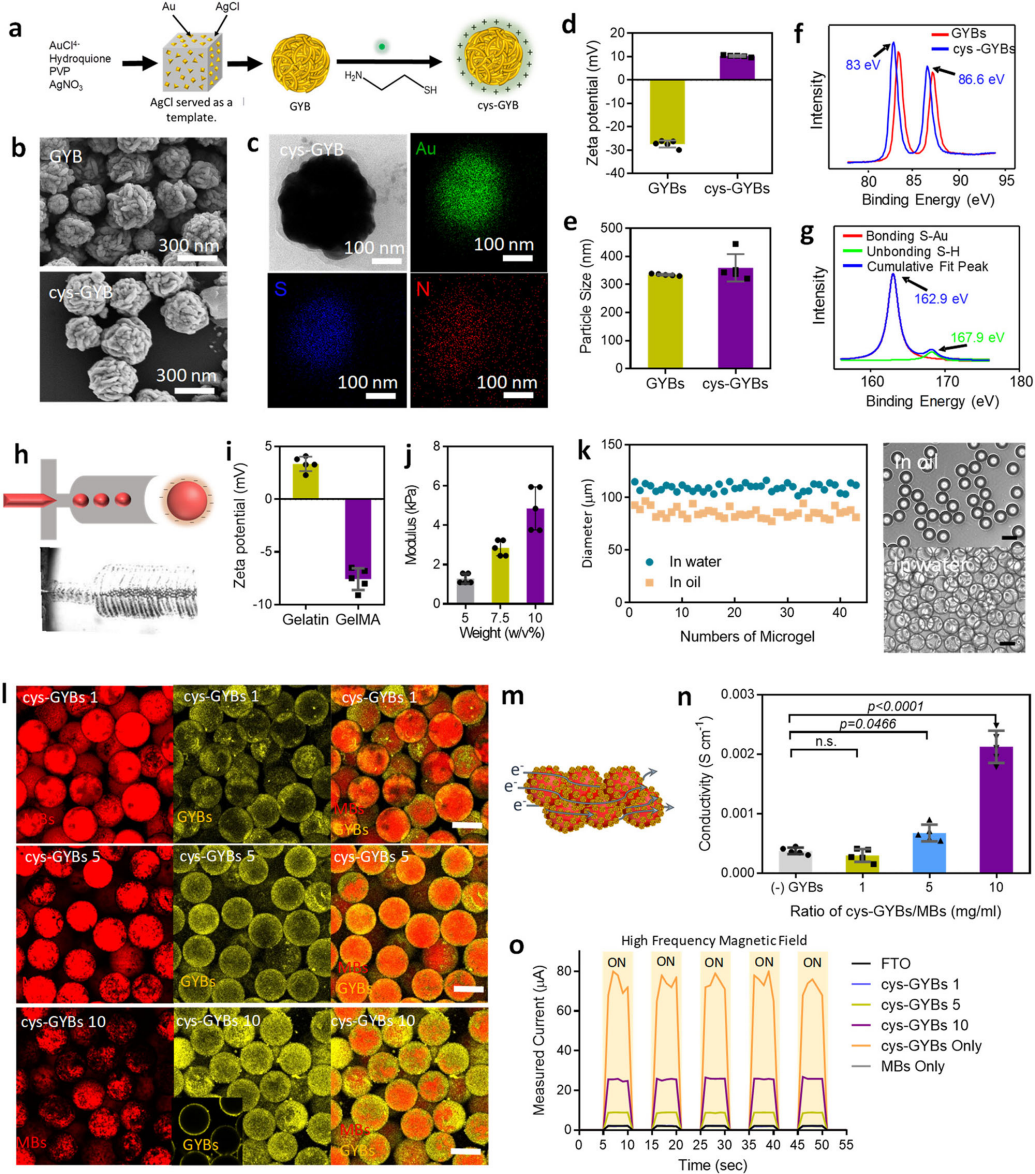
Preparation and characterization of conductive microporous hydrogel (CMH). **a** Schematic illustration of synthesis of cys-GYBs. **b** Scanning electron microscopy (SEM) images of GYBs and cys-GYBs. **c** TEM images of cys-GYBs and distribution of the elemental mapping of Au, S and N. **d**–**e** The surface charge of GYBs and cys-GYBs and zeta-potential measurements. Error bars represent mean ± s.d., *n*  =  5. HRXPS analysis of GYBs and cys-GYBs via (**f**) Au4f signal and (**g**) S2p signal. **h** Microscope image of the device generating GelMA droplets. **i** Zeta potential of gelatin and GelMA. Error bars represent mean ± s.d., *n*  =  5. **j** Compressive modulus of GelMA at various concentration. Error bars represent mean ± s.d., *n*  =  4. **k** Droplet size distribution of MBs in water and in oil. **l** Fluorescence images show the beads (red) with cys-GYBs (yellow) for visualization at different volume ratios. Scale bar: 100 μm. **m** Schematic of conductive microporous hydrogel. Blue line describes the proposed electron (e^−^) transfer passing through each CMH. **n** Electrical conductivity in the various hydrogels (*n* = 6, mean ± s.d., one-way ANOVA with Tukey’s multiple-comparison test). **o** Output currents on various materials generated by HFMF with on/off control.

To implement this design, electromagnetized GYBs and chemically modified gelatin-derived MBs were prepared. First, the GYBs were synthesized by a seed-growth method, where cubic silver chloride (AgCl) served as the sacrificing template, and the gold ligament was selectively deposited on the surface (Fig. 2a). Cubic AgCl was formed in advance by using a capping agent, namely, poly(vinylpyrrolidone) (PVP), and a silver nitrate ion-exchange approach in ethylene glycol. Then, the gold seed was deposited in the defects of the cube’s surface, which had a lower coordination number and stronger activity^31^. With a reducing agent (hydroquinone) and the surfactant PVP, porous GYBs with high densities of gold ligaments were formed within a few minutes. Finally, the AgCl templates were dissolved in the ammonia solution. Based on the scanning and transmission electron microscopy (SEM and TEM) results shown in Fig. 2b and Supplementary Fig. 1a, the GYBs with a mean diameter of 290 nm exhibited roughness. According to the Brunauer–Emmett–Teller (BET) analysis via nitrogen adsorption/desorption isotherms, GYBs had a surface area of ∼2.7 m^2^ g^–1^ and a pore size of ∼41 nm (Supplementary Fig. 1b). For surface charge modification of GYBs, cystamine was applied. Cystamine-modified GYBs (cys-GYBs) were obtained by the surface modification of cystamine (cys) through a thiol reaction on gold. After modification, the morphologies of the GYBs did not display apparent changes (lower row in Fig. 2b).

To further confirm the conjugation of cystamine, the presence of the Au, S, and N elements in the composite was evaluated by energy dispersive spectroscopy (EDS) mapping analysis (Fig. 2c); this analysis demonstrated the presence of the key elements of cystamine (N and S) on the GYBs. The zeta-potential measurement and particle size distribution results of the cystamine-modified GYBs are shown in Fig. 2d, e. The surface charge of the GYBs shifted from −28 mV to 10 mV when thiol ligands were introduced (Fig. 2d), and the average size of the particles increased from 334.5 to 359.4 nm (Fig. 2e). Variation of zeta potential and size of the cys-GYB with pH and the presence of media/ cellular proteins was also offered in Supplementary Fig. 1c to e. The gold−sulfur bonds between the thiol group of cysteamine and the GYBs were investigated by high-resolution X-ray photoelectron spectrometry (HRXPS); the chemical bond formation between Au and S shifted the peaks (Au 4f) of the GYBs to 83.0 eV and 86.6 eV (Fig. 2f), and the binding energy (S 2p) at 162.9 eV revealed Au–S covalent bonds (Fig. 2g).

To obtain MBs, gelatin methacryloyl (GelMA) was prepared by substituting amines of gelatin with methacrylamide in advance^32^. Through a microfluidic flow-focusing chip, high-throughput production and monodispersed GelMA MBs were formed (Fig. 2h). The zeta potentials of gelatin and GelMA were + 4.5 mV and −7.85 mV, respectively. The difference was caused by grafting methacrylamide to the amino groups of gelatins (Fig. 2i). This fabrication system could manipulate the mechanical properties of MBs by changing the polymer concentration, which affected cell proliferation and differentiation^33, 34^. The elastic moduli of 5 wt%, 7.5 wt%, and 10 wt% GelMA with a degree of substitution (DS) of 51% were ∼1.3, ∼2.8, and ∼4.9 kPa, respectively (Fig. 2j). In this study, 5 wt% GelMA with an elastic modulus of 1.3 kPa was used for further experiments since this value closely matched the modulus of cortex tissue^35^. After photocrosslinking, the narrow size distribution of GelMA droplets with an average diameter of 85 μm was collected while the oil phase flow rate was maintained at 20 μL min^−1^ and the flow rate of the GelMA solution was 1 μL min^−1^. Then, the MBs were washed and transferred into the water phase. In the water, the crosslinked MBs had a slightly larger average size of 100 μm (Fig. 2k) due to swelling. Finally, the MBs were stained with rhodamine B isothiocyanate (RITC) for tracking purposes (Supplementary Fig. 2).

The void space of CMH was evaluated by immersing CMH in a fluorescence solution (50 kDa of fluorescent dextran) to fill the void volume and then imaged by confocal laser scanning microscopy (CLSM). As shown in Supplementary Fig. 3a, the interconnected pores with similar patterns can be observed by fluorescence images. The result showed average void volume and pore of CMH are approximate 39 ± 11.3% and 45.43 ± 9.8%, respectively (Supplementary Fig. 3b). It implicates the advantage penetration of cells into the scaffold. The zeta potentials of the GelMA and cystamine-modified GYBs (cys-GYBs) revealed opposite charges at pH 7, suggesting electrostatic attraction under neutral conditions (Fig. 2d–i).

These adaptable properties were visualized by labeling cys-GYB and MBs with Cy5.5 and RITC, respectively. By simply mixing two particles at various ratios (cys-GYB 1 indicates that 1 mg of cys-GYB was mixed with 1 mL of MBs (0.61 g/mL)), the cys-GYBs (represented in yellow) were able to attach to the MBs rapidly (Fig. 2 l). While increasing the concentrations of cys-GYBs (5 and 10 mg), the coverage on the MBs was also increased (the cys-GYBs 5 and 10 groups in Fig. 2l). The fractional coverages of cys-GYB on the surface of microbeads were quantified by Imaris software. As shown in Supplementary Fig. 4a, the GYBs coverage on selected MBs were calculated by the overlapping fluoresce of GYBs and MBs, and the surface-exposed MBs were selected for evaluation. Based on the electrostatic attraction, the fractional coverage was increased from 29.5 ± 15.5% to 57.9 ± 16.0% with an increased ratio of GYBs/MBs from 1/1 to 10/1 mg/mL. Furthermore, the similar fractional coverage of 10/1 group at different periods under HFMF also revealed the stability of CMH after treatment (Supplementary Fig. 4b–d). Therefore, based on the stability and coverages, CMH with 10 mg/ml of cys-GYBs/MBs was applied in this study. Moreover, SEM images of the lyophilized CMH are presented in Supplementary Fig. 5. The amounts of GYBs were increased on the surface of MBs while increasing the GYBs ratios.

The electrical conductivities of (-) cys-GYBs (MB only), cys-GYBs 1, cys-GYBs 5 and cys-GYBs 10 were also investigated, and the electrical conductivity needed to be improved by the GYB conductors to enable the construction of a continuous electrical flow (solid blue line, Fig. 2m). As shown in Fig. 2n, weak conductivity was detected in the inefficient coverage of cys-GYB on MBs ((-) cys-GYBs and cys-GYBs 1). The cys-GYBs 10 group exhibited enhanced conductivity compared with all the other groups, and its conductivity was seven times higher than that of the (-) cys-GYBs group. The high conductivity of GYB 10 was potentially attributed to a large amount of cys-GYBs present in each MB.

To understand the penetration depth of HFMF, the eddy current on cys-GYBs induced by HFMF was performed on a 10 cm of fluorine-doped tin oxide (FTO) substrate (Supplementary Fig. 6), in which the wires were connected on both sides (non-current-induced area) to avoid the influence of wires-induced currents. While applying HFMF, output current was observed and depended on the concentration of cys-GYBs (Fig. 2o). The induced current by cys-GYBs group was about 78.5 μA, which was much higher than FTO alone group (approximately 2.1 μA)^36, 37^. In the CMH group, the currents could still reach 2.2, 9 and 25 μA in cys-GYBs/MBs ratio of 1, 5, and 10 groups, respectively. To evaluate the eddy current generated by the CMH, the compact gel was placed under a HFMF at a power of 3.2 kW and a frequency of 1 MHz. While applying an external HFMF to the CMH, the currents could immediately light up the light-emitting diode (LED), suggesting a serially connected circuit in this gel (Supplementary Fig. 6). Bright emission was observed in the cys-GYBs 10 group, whereas negligible intensity was observed in the other groups. These results indicated that electrical flows rely on the concentrations of the cys-GYBs adsorbed on the MBs.

### Rheological and self‐healing properties of CMH

**Fig. 3:**
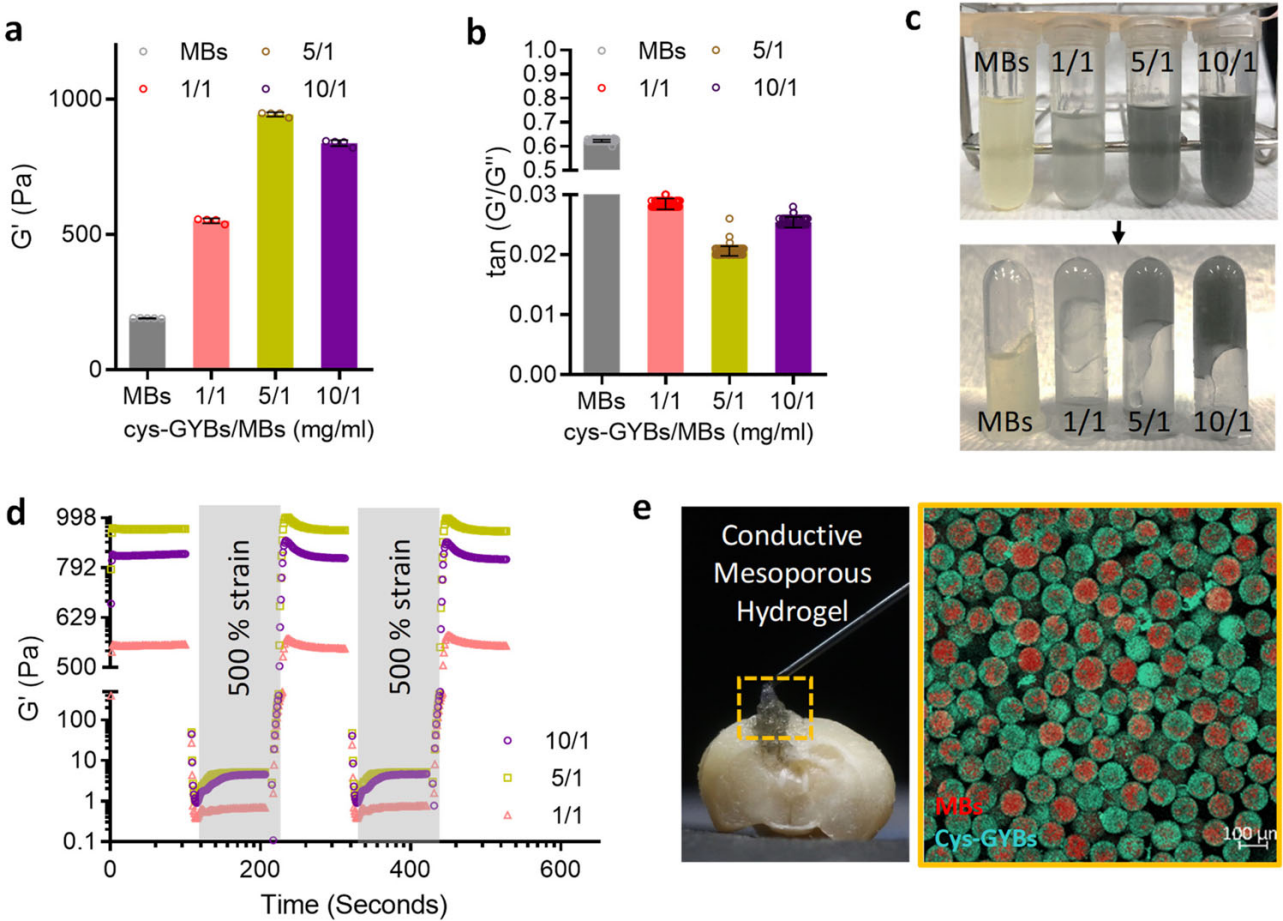
Rheological characterization of self-healing behavior of CMH. **a** Storage (G′) modulus and (**b**) tan (G”/G’) of CMH as a function of cys-GYBs-to-MBs ratio (mg/ml). Error bars represent mean ± s.d., *n*  =  5. **c** The gelation of CMH at various cys-GYBs-to-MBs ratios. **d** The damage-healing property of CMH demonstrated by the continuous step strain (1% strain→500% strain→1% strain) measurements at 37 °C. **e** CMH was modulable to macroscale shapes in a TBI cavity.

The gelation properties of CMH are also evaluated, in which the storage modulus (G’) of CMH was much higher than MBs alone. At a higher cys-GYBs-to-MBs ratio (mg/ml) of 5, the storage modulus (G′) of the CMH increased to about 990 Pa and gradually decreased to 800 Pa at the ratio of 10. This phenomenon can be explained by the lower electrostatic force between positively charged cys-GYBs and the insufficient negative surface of MBs, which was shielded by high concertation of cys-GYBs (Fig. 3a, b). Based on these interactions, the resulting CMH was much less flowable than MBs group (Fig. 3c). The reversibility of the noncovalent interparticle bonds endows these composite colloidal gels with self-healing properties. The storage (G′) and loss (G″) modulus were measured after multiple cycles of shear-induced gel network destruction under sequential strains of 1% and 500%. Remarkably, the CMH groups revealed an exceptional self-healing capacity since gel elasticity repeatedly recovered after network destruction (Fig. 3d). Furthermore, the injectable CMH scaffold can be modulable to unregularly cavity and maintain the microstructure after injection (Fig. 3e).

### In vitro electromagnetized GYBs stimulate neuron differentiation

**Fig. 4:**
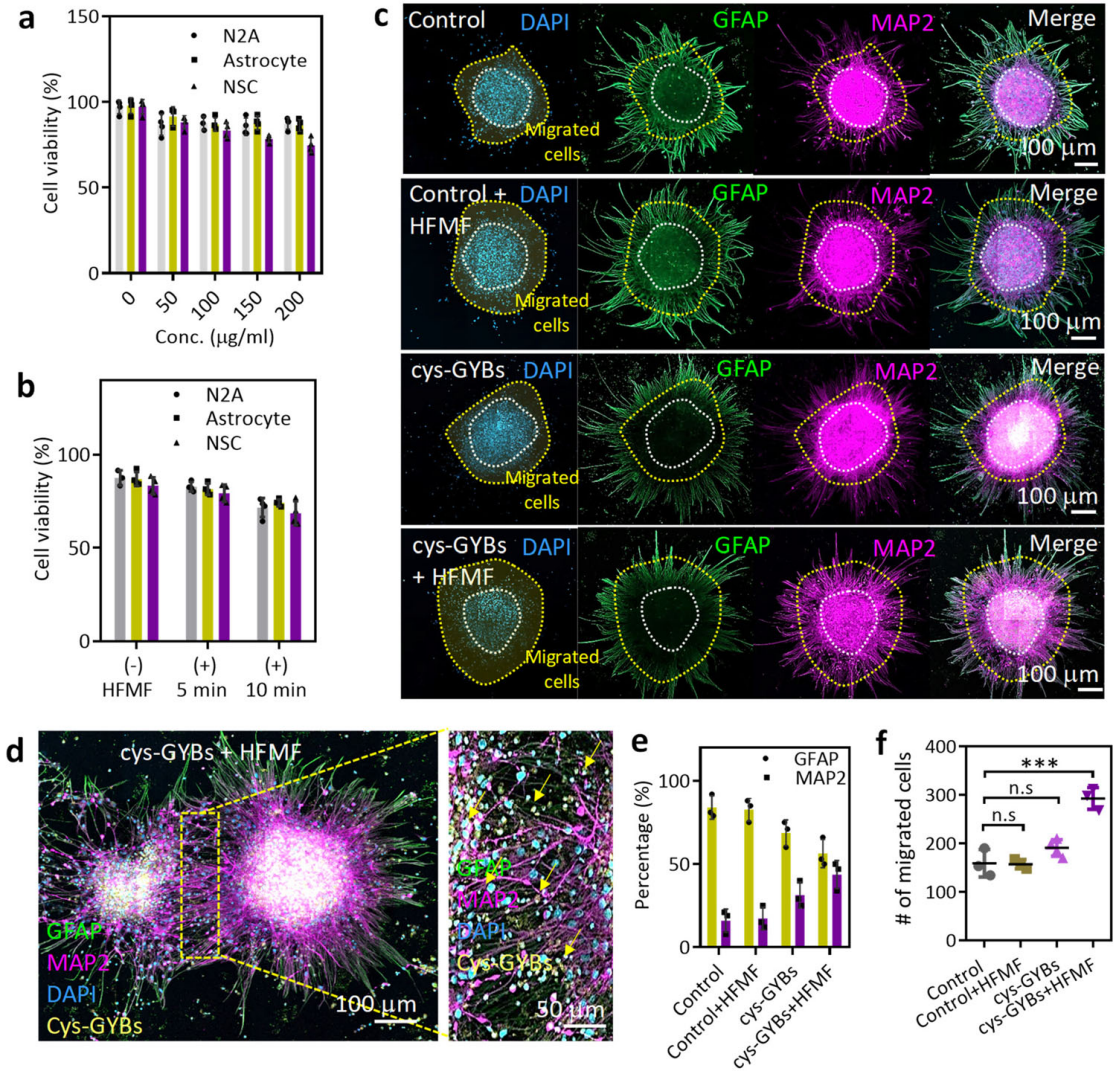
Electromagnetized cys-GYBs stimulate neuron differentiation. **a** Cell viability of NSCs, N2A cells and astrocytes treated with different concentrations of cys-GYBs. Error bars represent mean ± s.d., *n*  =  5. **b** Cell viability of NSCs, N2A cells and astrocytes treated with 100 μg/ml cys-GYBs with or without HFMF exposure. Error bars represent mean ± s.d., *n*  =  5. **c** Fluorescence photomicrographs showing the phenotypes of the cells that differentiated from embryonic cerebral cortical neurospheres after 7 days in culture. Anti-MAP-2 (purple) and anti-GFAP (green) antibodies show the immunoreaction of differentiated neurons and astrocytes, respectively. **d** Magnified images of the GYB + HFMF group. **e** Quantification of the percentage of differentiation into neurons and astrocytes from neurospheres. Error bars represent mean ± s.d., *n*  =  5. **f** Quantification of the number of migrated cells in the migrated zone. The cells were cultured under serum-free conditions at 250 neurospheres per cm^2^ for 7 days. Data represent the mean ± SD (*n* = 4 per group). ***p < 0.005 compared with the PBS group by one-way ANOVA with Tukey’s multiple-comparison test. Scar bars: 100 mm.

The effects of the GYBs treated with or without a HFMF on biocompatibility were evaluated by coculturing the GYBs with neural stem cells (NSCs isolated from ED 14−15 Wistar rat embryos), neuroblastoma cells (N2a cells), and astrocytes at different concentrations. Without the application of an HFMF, the cell viability was lowest in NSCs cultured with different concentrations of GYBs, but these cells still exhibited 81% viability at the concentration of 200 μg/ml, indicating low toxicity and biocompatibility (Fig. 4a). Compared to the NSCs, the N2a cells and astrocytes exhibited higher cell viabilities (greater than 90%) when cocultured with various concentrations of GYBs. This good biocompatibility was explained by the excellent stability of gold, which is a bioinert metal. Next, an HFMF with a power of 3.2 kW and frequency of 1 MHz was applied to the GYB-treated cells (Fig. 4b). There were only slight effects on cytotoxicity in the cells cultured with the GYBs under an HFMF at 5 min of treatment. When the treatment time was increased to 10 min, the cell viability of the three cell lines remained at ∼75%.

The use of electrical stimulation for manipulating the proliferation and differentiation of neural stem cells into neuronal cells by activating intracellular signaling pathways and intracellular microenvironments has also attracted great attention^28, 29^. To explore the effects of eddy currents on GYB-treated NSCs, NSCs were cultured with GYBs and treated with a HFMF for 5 min (Supplementary Fig. 7). At 4 days posttreatment, compared to the cells treated with GYBs or HFMF alone, the NSCs treated with GYBs and HFMF together exhibited obvious sprouting, indicating the current-induced neural-related cell differentiation of NSCs. Furthermore, the expression of a neuron marker (microtubule-associated protein 2, MAP-2) and astrocyte marker (glial fibrillary acidic protein, GFAP) was analyzed to assess the differentiation fate. In Fig. 4c, in the control group (without application of GYBs+HFMF) or the group treated with HFMF alone, the neural stem cells preferred to differentiate into astrocytes rather than neurons. However, compared with the other groups, the group treated with cys-GYBs exposed to a HFMF (The power was 3.2 kW with a strength of 4 kA/m at a frequency of 1 MHz) exhibited efficient neuron differentiation with an increase in the number of MAP-2-positive neurons. GYBs were observed in the neurospheres and sprout cells, and most GYBs were located close to the nuclei (Fig. 4d and Supplementary Fig. 8). The results indicated that the cys-GYBs demonstrated good affinity and low cell toxicity. These results also demonstrated that the electromagnetized GYBs promoted stem cell differentiation and neurite outgrowth.

By applying CHM, it was also able to promote stem cell differentiation upon a 5 min of HFMF treatment per day. After 7 days, similar to cys-GYBs treatment, the CLSM images of CMH-treated NSCs indicated the stem cell differentiation and neurite outgrowth (Supplementary Fig. 9a). After the quantification, CMH consisting of 10 mg of cys-GYBs and 1 ml of MBs promoted approximately 30.33% of NSCs differentiation into neuronal cells when compared to the control group, indicating the effective stimulation of electromagnetized cys-GYBs on CMH (Supplementary Fig. 9b).

To understand the effects of the HFMF on the differentiation fates of cells, MAP-2- and GFAP-positive cells were evaluated. The percentages of neurons in both groups treated with GYB were higher than those in the control and control+HFMF groups; a 2.5-fold increased neuronal differentiation was observed in the cys-GYBs+HFMF group compared to the control group (Fig. 4e), suggesting the positive influence of electromagnetized cys-GYBs on successful neuronal differentiation. Furthermore, the mature NSCs that migrated from the neurospheres were evaluated by calculating the number of migrated zones (Fig. 4c). The area between the inner neurosphere and outer line represented a cell migration zone. These results showed an approximately 1.9-fold increase in MAP-2-positive neuronal cells migrating away from the neurospheres after treatment with GYBs+HFMF (Fig. 4f). The behaviors were potentially attributed to the particles, and the induced currents substantially promoted the interactions between cells and the cell/matrix in the neurospheres by activating intracellular signaling pathways, increasing self‐renewal ability, and accelerating differentiation^28, 29, 38^.

The ability of the CMH scaffold to promote cell penetration and proliferation in vitro was estimated based on the cellular morphology and proliferation within the gel using two cell types (astrocytes and fibroblasts). For imaging purposes, MBs stained with rhodamine B isothiocyanate (RITC) are shown in red, and F-actin staining of the cell cytoskeleton is shown in green. In Supplementary Fig. 10, both astrocytes and fibroblasts directly adhered to and proliferated in the scaffold within 4 days without additional steps to promote protein adhesion, demonstrating the continued proliferation and network morphology in the CMH due to innate cytocompatibility. On the 7th day, higher portions of cells infiltrated into the CMH.

### In vivo study of regenerated nerve in TBI

**Fig. 5:**
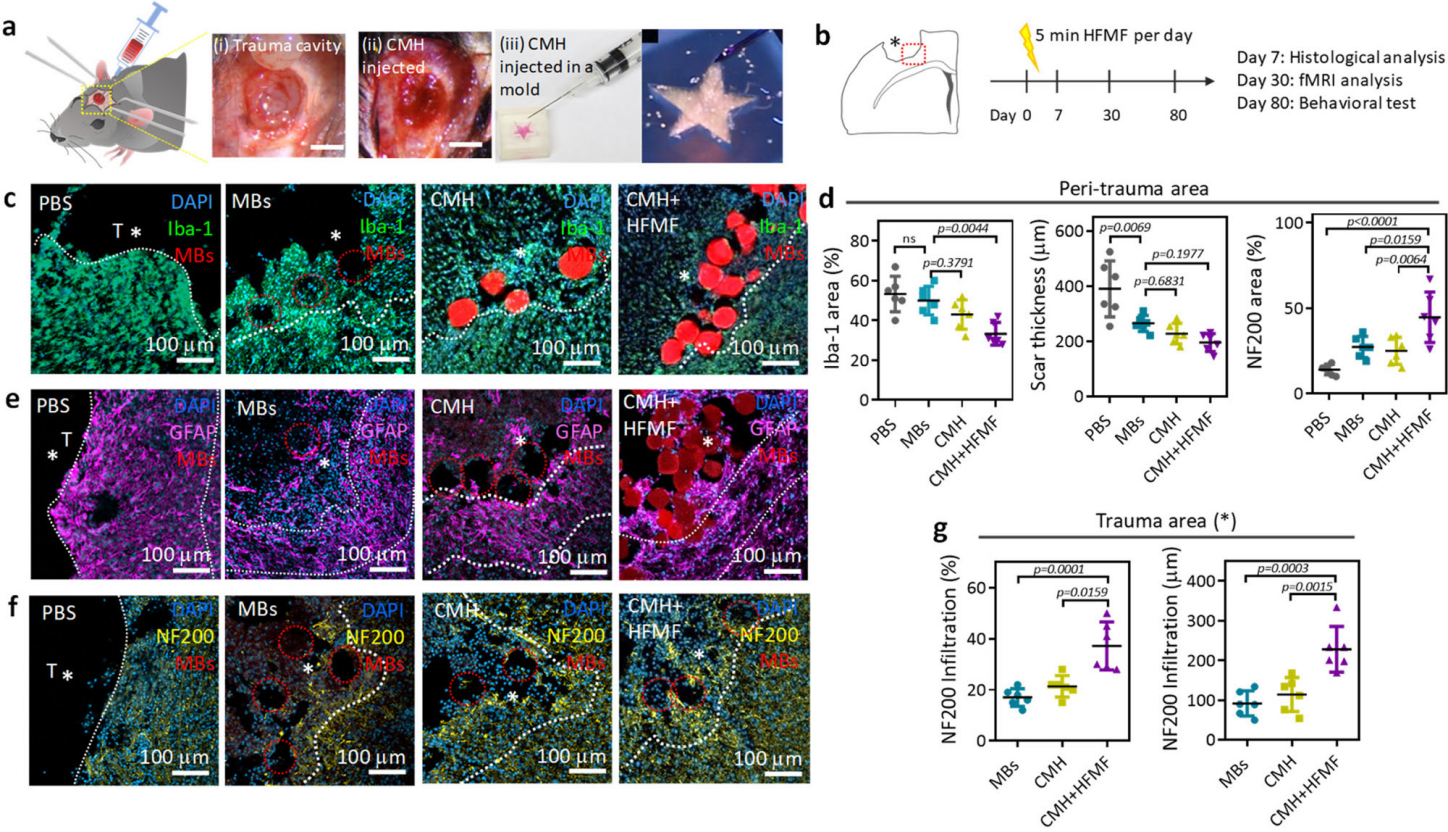
Animal study. **a** Images of the TBI cavity before and after implanting the CMH scaffold (left panel). Images of CMH injected into a star-shaped mold via a syringe (right panel). **b** Schematic of the in vivo experimental timeline, including the sacrifice and analysis time points. **c** Fluorescence images of Iba-1 immunostaining after treatment with PBS, MB, CMH, and CMH + HFMF. **d** Analysis of Iba-1, scar thickness and NF200-positive responses at peri-trauma areas (*n* = 6, mean ± s.d., one-way ANOVA with Tukey’s multiple-comparison test). Fluorescence images of (**e**) GFAP and (**f**) NF200 immunostaining after various treatments. **g** Analysis of the NF200-positive responses and infiltration distance in the trauma area. (*n* = 6, mean ± s.d., one-way ANOVA with Tukey’s multiple-comparison test).

Having demonstrated cell penetration and ex vivo NSC differentiation, CMHs were then applied to an animal model. In vivo, TBI was established by removing brain tissue by using a flat-end puncher (2 mm in diameter) to generate an injury with a depth of 1.5 mm (Fig. 5a). Then, CMHs were implanted into the brain cavity by a syringe (Fig. 5a(i–ii)). CMH was also injected into a star-like mold that the CMH could successfully and spontaneously fill, even in the sharp corners (Fig. 5a(iii)). Figure 5b shows the in vivo treatment course: to analyze immune responses, the time point at 7 days postinjury coincides with the recruitment of other peripheral immune cells and the local activation of microglia and astrocytes^39, 40^; the evaluation of angiogenesis measured at 30 days postinjury was due to the vessel maturation period and a chronic period in tissue as well as the behavioral recovery in traumatic brain injury. For the HFMF-treated group, a magnetic field at a power of 3.2 kW and frequency of 1 MHz was applied for 5 min per day until the mice were sacrificed. At 7 days postsurgery, the inflammatory responses at the peri-trauma area of the TBI were evaluated by assessing microgliosis, astrogliosis, and neuroinflammation after the different treatments. The trauma boundaries were defined by the intact and fluorescence intensity of the tissues (Supplementary Fig. 11). First, the microglial responses were assessed by ionized calcium-binding adaptor molecule-1 (Iba-1) staining in Fig. 5c (The presence of the gel is inferred by the voids left behind in the section, in which the gel was not observed due to the tissue slicing, washing and immune staining processes.) The CMH-treated group exhibited a weaker fluorescence intensity of Iba-1 than treatment with PBS-treated group, indicating that the CMH could reduce the inflammatory response in the traumatic injury^17^. Compared to the transplantation of nonporous hydrogels, the transplantation of porous materials into damaged tissue enhances the penetration of glial fibrillary acid protein (GFAP) and reduces wound scar thickness^16^. In addition, after applying a HFMF to the CMH at the TBI site, the regions occupied by microglia were further decreased in both the peri-trauma areas. This observation is reminiscent of modulating intracellular signaling pathways via electron stimulus, which reduces inflammatory cytokines such as interleukin‐17A and the infiltration of CD3-positive cells^41^. The quantification of the area occupied by microglia is also provided in Fig. 5d, where ∼32.5% of the peri-trauma area was positive for microglia in the CMH + HFMF group. However, higher microgliosis of ∼54.4%, ∼50.0%, and ∼43.9% was observed in the peri-trauma areas in the PBS, MBs, and CMH groups, respectively.

In addition to immune cell infiltration, activated resident microglia cells produce pro-inflammatory cytokines, such as IL1β, IL6, IL12, and TNFα, resulting in increased inflammatory activity and secondary cell death^42^. IL-6 is a pro-inflammatory cytokine, which is usually used to elevate the inflammatory responses after tissue injury or an inflammatory stimulus^43^. After 7 days postinjection, CLSM images in Supplementary Fig. 12a displayed the intensity of interleukin-6 (IL-6) expression at the peri-trauma area treated by MBs, where CMH and CMH + HFMF were lower than that treated by the PBS group (Supplementary Fig. 12b), indicating the reduction of the inflammatory response. This trend is consistent with the tendency of IBa-1 staining (Fig. 5d). These results support an at the present underexplored geometric component to immune stimulation with what has been observed in other microporous scaffolds that are cast ex vivo and implanted in vivo^17^. The materials corroborated the greater ease of cell mobility and better infiltration of inflammatory cells. Furthermore, the MB scaffold and its surrounding tissue exhibited a lower number of microglia when compared with the PBS group.

After the evaluation of microgliosis, astrogliosis and astrocytic scar thickness were estimated by glial fibrillary acidic protein (GFAP) staining (Fig. 5e). In each group of animal study, six C57BL/6 mice (Female, 7 weeks) were used (n = 6). 5 slices per animal and 3 ROIs in traumatic regions were randomly chosen and calculated as below images (Supplementary Fig. 13a). The astrocytic scar thickness in the various MB groups was decreased compared to that in the PBS group. The scars in the MBs, CMH, and CMH + HFMF groups were 264.4, 228, and 191.8 μm thick, respectively. In contrast, the scar was approximately 400.8 μm thick in the PBS group (Supplementary Fig. 13b). The reduction in scar thickness was possibly attributed to the microporosity in the hydrogel, which provided interconnected channels and void spaces for cell infiltration^18^. Furthermore, this result showed that the MBs or CMH scaffold sustained astrocyte infiltration without the assistance of additional biological or chemical cues. The infiltration of regenerative astrocytes into the trauma area could offer benefits for tissue recovery^16^. We demonstrated that both astrogliosis and microgliosis were decreased in the TBI cavity via porous CMHs, resulting in reduced scar thickness and decreased reactive microglia.

Following the investigation of the anti-inflammatory response, the axonal infiltration distance and area identified by an axonal marker (Neurofilament 200, NF200) in the peri-trauma and trauma areas were examined (Fig. 5f). At 7 days postinjury, the percent of NF200-positive area in the peri-trauma region was only ∼14.4%, indicating the large portion of lost neurofilaments (intermediate filaments in the cytoplasm of neurons). The TBI cavity was devoid of neurofilaments at this time point. We found that axon numbers (NF200) increased in the peri-trauma area following injection of the MBs and CMHs, and an increase was observed in CMH + HFMF at 7 days postsurgery compared with the PBS group. The average areas of the neurofilament regions in the peri-trauma area following treatment with the MBs and CMHs were ∼27.2% and 25.6%, respectively, indicating a negligible difference in the NF200-positive area between the two groups (Fig. 5d). In the trauma cavity, the axonal infiltration was observed in the MB- and CHM-treated groups but not in the PBS-treated group. These results suggested that the fewer astrocytes and macrophages/microglia in the CMH promoted a pro-reparative microenvironment at an early stage, facilitating further axonal sprouting. Remarkably, in the CMH + HFMF group, the NF200-positive area (∼47.3%) was increased by almost 4-fold compared with that in the PBS group (Fig. 5d). CMH + HFMF treatment increased in axonogenesis in the peri-trauma area and promoted neuron filament infiltration into the trauma site with an average infiltration length of ∼237.6 μm (∼32 μm in the PBS group) in Fig. 5g. To the best of our knowledge, the substantial numbers of NF200-positive cells observed infiltrating the TBI cavity have rarely been reported in previous works. The mechanism was potentially caused by electrical stimulation-induced activation of neuronal differentiation, nerve repair, and neurite outgrowth through the upregulation of calcium signaling and phosphorylation of cAMP response element binding (CREB). Such stimulation influences various cell types and activates intracellular signaling to drive neurogenesis^44^.

### In vivo cell infiltration and vascular formation

**Fig. 6:**
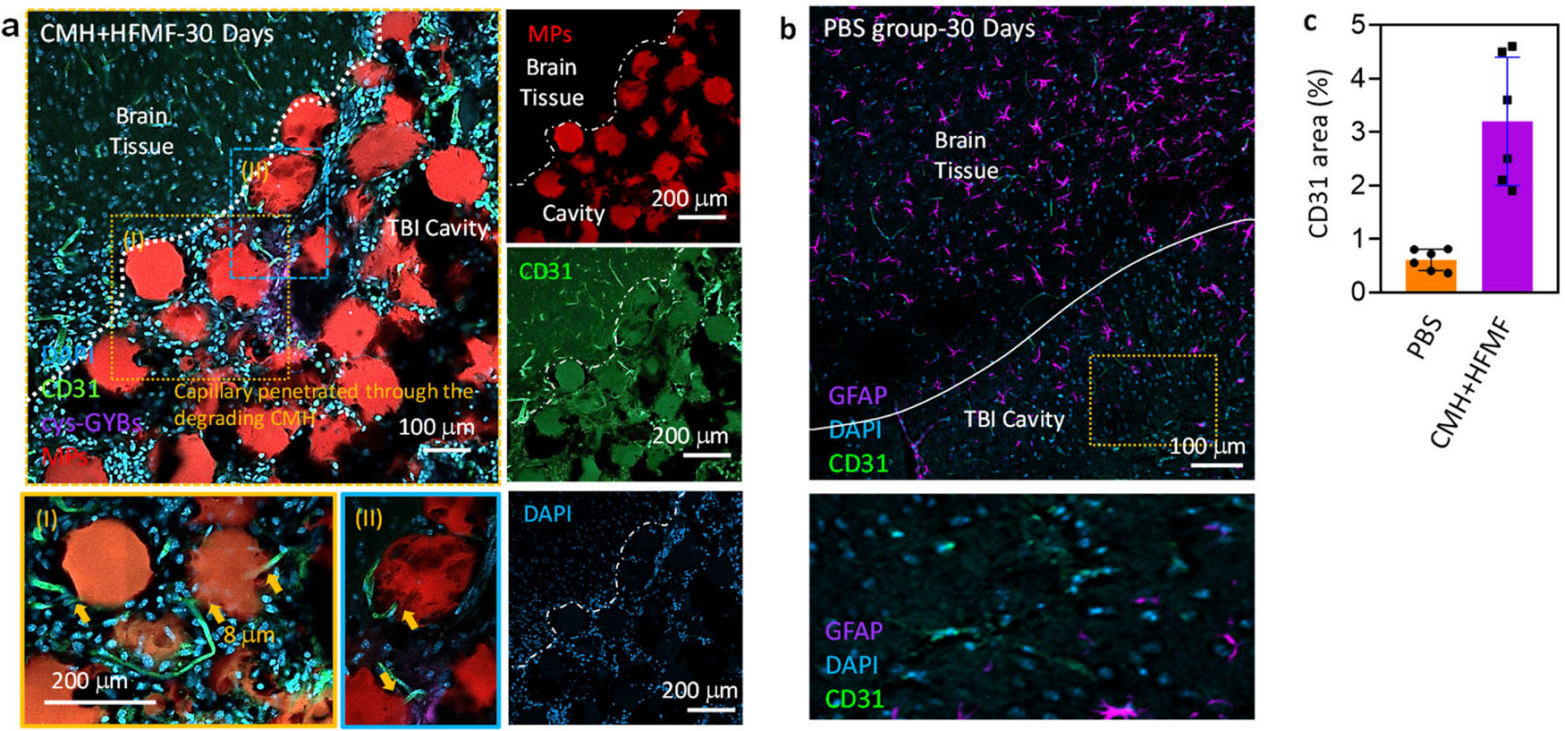
Endothelial cells (ECs) infiltrated and ingrowthed within channel network hydrogels from 30 days implantation in mouse. Fluorescence images of blood vessels (CD31) in green), nucleic (DAPI) in blue and MBs (RITC) in red around the trauma site at 30 days postsurgery. **a** ECs lining a microchannel network in vivo after treated by cys-GMH + HFMF group (I and II: a cross-section view of EC-lined channel). Scale bar = 150 µm. **b** The fluorescence images of control group. **c** Quantification of CD31 area of images at TBI cavity Error bars represent mean ± s.d., *n*  =  6.

Cell infiltration and vascular formation in the interconnected pores of hydrogels in damaged tissue are also critical factors that regulate tissue regeneration^18^. To monitor vessel infiltration and formation at the TBI site after CMH + HFMF treatment, the vessels were stained with the biomarker CD31 at 30 days postsurgery. In Fig. 6a, the formation of vascular structures was observed in the CMH, and some vessels even infiltrated into the interconnected pores of the CMH. Following the closer observation, the formation of capillaries surrounded the microchannels of the CMH and penetrated the MBs while the MBs were degraded (Fig. 6a). Well-formed blood vessel networks with endothelial cell infiltration lengths of ∼437.6 μm and many cells were also observed in the CMH. This evidence suggests that improved cell migration and vascular-like network formation inside the composite leads to axonogenesis in the TBI cavity. However, only a few CD31 signals were observed in the trauma cavity in the control group (Fig. 6b). The quantification of the resulting fluorescence area of CD31 at the TBI cavity was also exhibited in Fig. 6c. Furthermore, to investigate the effect of vascular cells on scar thickness and astrocyte infiltration, the thickness of the GFAP^+^ layer surrounding the TBI cavity and the infiltration distance of the GFAP+ cells starting from the cavity border were evaluated. As expected, the PBS group exhibited obvious scar thicknesses, but the CMH + HFMF-treated group successfully mitigated scar formation (peri-trauma GFAP^+^ area) after 30 days (Supplementary Fig. 14). Moreover, newborn cells stained with BrdU were also observed in the trauma cavity after 30 days (Supplementary Fig. 15).

### In vivo BOLD-fMRI evaluation

**Fig. 7:**
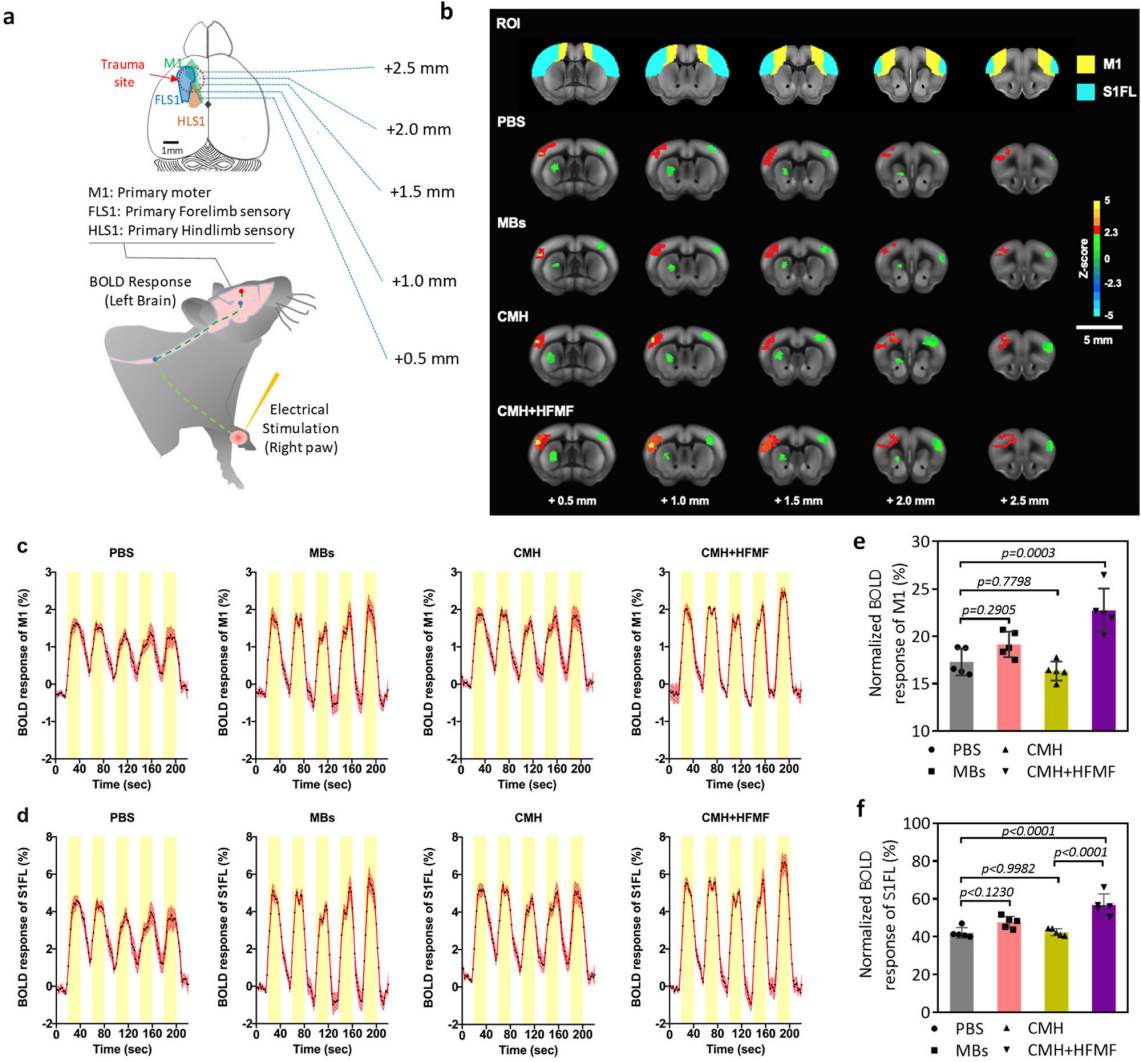
Blood-oxygenation level-dependent (BOLD) fMRI activation maps during contralateral forepaw stimulation. **a** Illustration of the experimental design. Activation was triggered by electrical stimulation of the right forepaw 30 days after TBI. **b** Comparison of activation Z-maps obtained by group-level BOLD contrasts in the treated groups of PBS (*n* = 6), MBs (*n* = 6), CMH (*n* = 6) and CMH + HFMF (*n* = 6), respectively, superimposed on corresponding high-resolution RARE T2 images. Visualization of activation Z-maps displayed at z- threshold free and their activity in voxels within the red boundary reached the significant level threshold at Z > 2.3 (*p* < 0.05). Mean time courses of relative BOLD responses in contralateral (**c**) M1 and (**d**) S1FL in response to forepaw stimulation each group. The intervals of electrical stimuli are represented by yellow boxes with five repetitions. Data are represented as mean ± SEM. The average number of activated pixels across all slices representing ipsilateral (**e**) M1 and (**f**) S1FL, respectively. (*n* = 6, mean ± s.d., one-way ANOVA with a Tukey’s multiple-comparison test).

Blood-oxygenation level-dependent functional magnetic resonance imaging (BOLD-fMRI), a noninvasive neuroimaging technique for evaluating the ratio of oxygenated hemoglobin (blood flow) and brain function, was used to investigate the corresponding neurophysiological recovery 30 days postinjury. The detailed description of BOLD-fMRI acquisition and data analysis were presented in Supplementary Note 11. The evoked BOLD responses were detected in the primary somatosensory cortex of the forelimb (S1FL) and primary motor cortex (M1) while applying electrical stimulation to the contralateral forelimb (Fig. 7a). The stimulation paradigm of the fMRI experiment consisted of a block design starting with a resting period of 20 s (baseline), followed by five cycles of a 20-s stimulus OFF period and a 20-s stimulus ON period each, including 5 stimuli blocks and 6 control blocks without forepaw stimulation. Electrical stimulation was obtained by delivering the biphasic electrical current with a 1 mA amplitude, 25 μs duration and 12 Hz frequency applied in a block design (Supplementary Fig. 16). The group-level BOLD fMRI activation Z-maps (the significant level was set at Z > 2.3 (*p* < 0.05), positioned at bregma +0.5 to +2.5 mm) were overlaid on the corresponding high-resolution MRI anatomical images with a rapid acquisition with refocused echoes (RARE) T2 sequence (Fig. 7b). Our results demonstrated that the CMH + HFMF group presented a significant increase in the number of activated voxels with the positive BOLD signal in the M1 and S1FL regions in and around focal injuries. For further quantification of corresponding neurological outcomes in the different treatment groups, the BOLD response magnitudes of functional activations also were examined in M1 and S1FL regions. For group comparison of BOLD time courses, the corresponding time courses of evoked BOLD signals obtained from 4 treatment groups showed their averaged contrast magnitudes on the orders of 2–3% in M1 cortex and 4–7% in S1FL cortex (from the resting signal level under stimulus OFF) as shown in Fig. 7c, d, which there were sufficient statistical powers (>0.8) and medium to large effect sizes each group to detect BOLD signal changes between stimulus ON and OFF with enrolling enough number of animals used (Supplementary Table 1). However, there was no significant response delay relative to the electrical stimulus time course observed in each treatment group. The low temporal resolution of our fMRI scan protocol with the repetition time (TR) of 2 sec could not provide an accurate representation of the BOLD signal response delay. In order to achieve better comparison of functional recovery across treatment groups, the normalized BOLD responses were determined by the ratio (%) of the evoked BOLD magnitude over the injured brain region on the left hemisphere to those over the equivalent non-injured on the right hemisphere. The PBS-treated groups exhibited lower normalized BOLD responses in both M1 and S1FL cortical regions than the other groups. In addition, a significant increase in normalized BOLD responses was found in CMH + HFMF-treated groups compared with other groups (Fig. 7e, f), resulting from the promotion of neuroplasticity to produce a significant functional recovery of the corticostriatal and corticolimbic tracts. During the late phase of recovery from TBI, residual neural circuits were reconstructed, perhaps partially compensating for the lost parenchyma function^45^. Therefore, the CMH + HFMF-induced electrical neuromodulation might accelerate adaptive neuroplasticity or repair of injury neural circuits to enhance the functional recovery after TBI^46, 47^.

### In vivo motor and somatosensory in brain functions

**Fig. 8:**
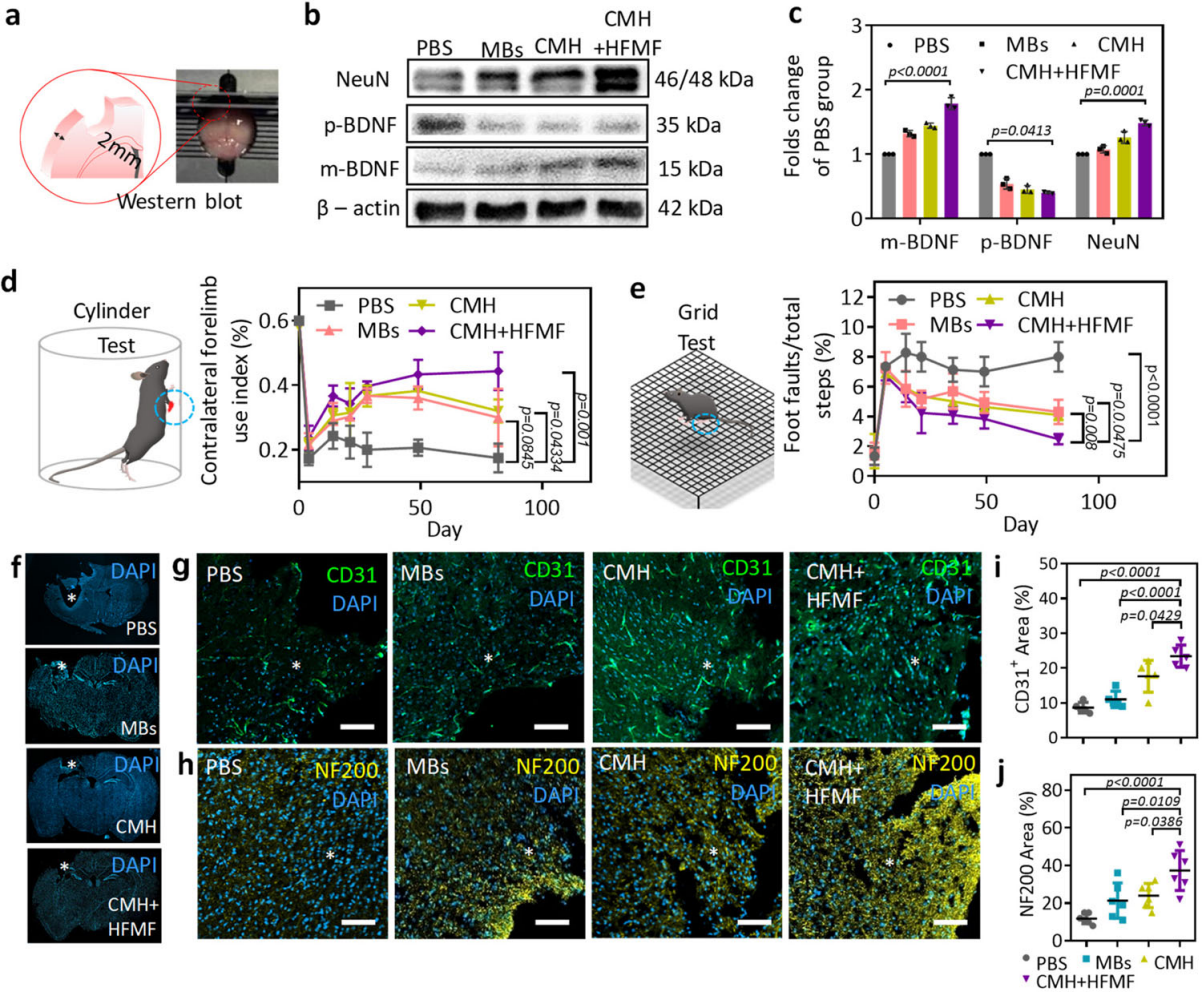
Western blot analysis and post-TBI neurological recovery and axonal sprouting. **a** Tissue slice of trauma area of ipsilateral brain slice with 2 mm. **b** Western blot analysis of brain. β-actin was selected as the internal control with a protein weight of 42 kDa; the values of m-BDNF and p-BDNF were 15 and 35 kDa, respectively. NeuN was 46 and 48 KDa, respectively. **c** The quantitative results of proteins are expressed as the ratio of densitometries of m-BDNF, p-BDNF and NeuN to β-actin bands. Error bars represent mean ± s.d., *n*  =  6, one-way ANOVA with a Tukey’s post-hoc test. **d** The cylinder test to evaluate the dexterity of their contralateral forelimb. Error bars represent mean ± s.d., *n*  = 6, one-way ANOVA with a Tukey’s post-hoc test). **e** The grid test for the contralateral hindlimb normally sensitive to post-TBI lateralized impairments. Error bars represent mean ± s.d., *n*  =  6, one-way ANOVA with a Tukey’s post-hoc test). **f** Representative brain images illustrated cavity of tissue loss (white circle) in mice at 82 days postinjury. Fluorescent images of (**g**) endothelial cells (CD31) and (**h**) axonal neurofilaments (NF200) around the TBI site (*) at 60 days postinjury. Quantification of the (**i**) vessels (CD31) and (**j**) axonal area (NF200) in the ipsilateral peri-trauma cortex at 60 days after TBI. Error bars represent mean ± s.d., *n*  = 6, one-way ANOVA with a Tukey’s post-hoc test). Scar bars: 100 μm.

To verify the functional recovery related to neuronal protection due to the secretion of neuronal nuclear protein (NeuN) and BDNF from the surviving neurons after TBI, the levels of these proteins in the peri-trauma zone of the injured brain were examined by western blot (Fig. 8a, b). The western blot analysis conducted at 8 days postsurgery was due to the increased ProBDNF protein level and the decreased BDNF level in the cortex of injured brain within the first week^48^. The level of the NeuN protein was used as an index of the level of mature neuron expression. Furthermore, proBDNF (pBDNF) and mature BDNF (mBDNF) have opposite effects on the regulation of repair processes in the central nervous system; pBDNF initiates neuronal apoptosis, and mBDNF affects the survival, differentiation and growth of neurons and promotes neuroprotective activity and synaptic plasticit^45^. The results showed an increase in the protein expression of pBDNF following TBI without any treatment (PBS group), and this group also exhibited neuron loss, i.e., low expression of the NeuN protein. Furthermore, both the protein levels of NeuN and mBDNF in the peri-trauma zones were higher in the CMH + HFMF group than in the other groups 30 days postinjury. The quantified intensity of mBDNF and NeuN in the CMH + HFMF group was 1.8-fold and 1.5-fold higher than that in the PBS group, respectively (Fig. 8c). In fact, the higher expression of mBDNF in response to electrical stimulation leads to the specific activation of neuronal protection, decreases mature neuron loss and reduces apoptosis in the peri-trauma zone, thus enhancing neuron survival, neuroplasticity, and functional recovery. More interestingly, CMH or MBs also improved neuron protection by NeuN and BDNF.

We further studied the long-term recovery of motor and somatosensory deficits in mice following treatment with CMH and HFMF. Limb responses were evaluated through two behavioral tests, i.e., cylinder test/asymmetry scores (Fig. 8d, left) and grid test/foot fault (Fig. 8e, left). The assessments reflecting the dexterity of the contralateral forelimb and hindlimb determined the motor-control patterns after TBI. The neurobehavioral status of the mice was evaluated on days 4, 14, 21, 28, 49, and 82 after the mice were treated with PBS, MBs, CMH and CMH + HFMF. In Fig. 8d, a cylinder test was performed to estimate the forelimb function by evaluating the balance of the animals touching the sidewall of the cylinder. As the results show, following a TBI injury in the forelimb motor area, the function of the impaired forelimb was obviously decreased, and unbalanced limbs were dragged along the wall. For all the groups, the gait and dexterous forelimb use were minimal at day 4, and some recovery of the infarct was observed at day 14. When no treatment (PBS group) was applied, the recovery of the forelimb motor activity was weak for 82 days because the TBI continued to deteriorate. The MB- and CMH-treated groups exhibited an improvement within 49 days, but motor recovery slightly declined at 82 days posttreatment. The potential reason of the decline in motor function recovery for CMH group at 82 days might be caused by no continuous treatment of the brain trauma, leading the astrocytic and fibrotic scars impeding recovery in the TBI cavity and resulting in cerebral atrophy in the motor/sensory cortex. Compared to CMH + HFMF group, the lower expression of mBDNF in CMH indicated the moderate neuronal protection and enhanced neuron survival in the peri-trauma zone. Therefore, it is difficult to establish a long-lasting repair response that includes angiogenesis and neurogenesis in the damaged tissue in the brain. Notably, the CMH + HFMF group consistently improved in the contralateral forelimb throughout the period. The index from 0.22 to 0.45 after 21 to 82 days postinjury indicated good motor recovery (Fig. 8d).

In the grid test, the CMH + HFMF-treated group showed the recovery with a decrease in the ratio of foot faults and total steps from 7.1% to 2.1%. However, the MBs and CMH groups exhibited no significant functional improvements in the grid walk task at the same time point 35–82 days postinjury (Fig. 8e, right). The results were also consistent with the anti-inflammatory effects and the expression of neuronal proteins.

At 82 days posttreatment, the TBI mice were sacrificed, and then, the brains were harvested. In the PBS group, the trauma cavity revealed a reduction in cortical volume (Fig. 8f). The cavity observed in the CMH + HFMF-treated group was smaller than PBS group. To further evaluate the effects on the symmetry and size of the ipsilateral ventricle, MRI anatomical images at the coronal level corresponding to the injury site were also evaluated 30 days postinjury (Supplementary Fig. 17). The symmetrical ipsilateral ventricle should be observed in a normal brain, and its condition indicates the degree of external traumatic injury. In the PBS-treated group, the MRI images showed asymmetrical and enlarged ipsilateral ventricles at the injury site, suggesting that the injury led to a considerable fibrotic response in the brain tissue and cerebral atrophy. In contrast, at 30 days postinjury, the mice treated with CMH + HFMF exhibited symmetrical ipsilateral ventricles and effectively reduced severe tissue loss, which prevented the deformation of TBI cavity.

To further prove that the observed functional recovery was associated with the axonal network due to angiogenesis and the infiltration of axons into the TBI site, markers of neurofilament (NF200) and blood vessels (CD31) were utilized to assess the density around the TBI site. As shown by western blot in Fig. 8b, BDNF at 8 days postsurgery was more highly expressed in the CMH + HFMF group and was expected to activate self-recovery in the injured brain via angiogenesis and neuroprotection. Corresponding to the expression of mBDNF, an increased fluorescence intensity of endothelial cells (CD31) was observed in the CMH + HFMF group at 82 days postinjury compared with the PBS, MBs and CMH groups (Fig. 8g), suggesting that the electric stimulus and porous gel improved angiogenesis at the TBI site. Furthermore, the NF200-positive area in the CMH + HFMF group was also higher than that in the other groups. These axons represented a network structure around the peri-trauma area in the CMH + HFMF-treated group (Fig. 8h). The quantitative mean intensity of the vasculature around the infarct was 2.7-fold higher in the CMH + HFMF-treated group than in the PBS group (Fig. 8i, the calculation of CD31^+^ area (%) was based on signal intensity). Notably, the neurofilament intensity in the CMH + HFMF-treated group was approximately 3.15-fold greater than that in the PBS group (Fig. 8j). These studies indicated that CMH + HFMF promotes the development of neurovascular brain tissue and functional recovery through angiogenesis and mBDNF production. The CMH + HFMF treatments also lead to a higher neuron survival rate and more axonal growth into a network of connections from adjacent brain tissues.

## Discussion

A versatile conductive hydrogel allowing the formation of interconnected pores in the TBI cavity was developed to serve as an injectable scaffold to reduce inflammation and activate mature brain-derived neurotrophic factor production in order to promote nerve tissue regeneration. These results were made possible by combining microbeads and electromagnetized gold nanoyarn balls (GYBs) with suitable mechanical support and noninvasive electric brain stimulation. The conductive hydrogel provides connected pores in traumatic brain trauma through which cells can penetrate to promote anti-inflammation and angiogenesis processes. Furthermore, an external high-frequency magnetic field (HFMF) that induces eddy currents on GYBs can promote neurite outgrowth both in neural stem cells (NSCs) and animals, lead to a 180% enhancement of mature BDNF (mBDNF) at the peri-trauma site. Integrating these functions, the hydrogel promotes an ∼2.5-fold and 3.15-fold increase in both peri-trauma vascularization and neuron survival in the trauma cavity, facilitating a restoration of brain functional connectivity (FC) in the corticostriatal and corticolimbic circuits. These conductive microporous hydrogels, which are characterized by tunable porosity and noninvasive brain stimulation via a temporally high-frequency magnetic field, create possibilities for hydrogel-mediated nerve therapy and potential use in clinics.

## Methods

### Ethics statement

The animal use and experimental protocols were approved by the Institutional Animal Care and Use Committee (IACUC) of National Tsing Hua University (Approval No. 10704).

### Synthesis and modification of gold nanoyarn-ball (GYBs)

The GYBs were prepared through a seed-growth method, where the cubic silver chloride (AgCl) was served as sacrificing templates and the gold ligament was selectively deposited on their surfaces. In brief, cubic silver chloride (AgCl) was prepared by mixing 70 μl of AgNO_3_ solution (100 mM) and 110 μl of HAuCl_4_ (40 mM) solution. Then, add the mixture into a 4.5 ml of 100 mM PVP solution by stirring. Finally, 160 μl of 28 mM hydroquinone solution was added slowly into the solution at room temperature without light irradiation. The solution instantly turned to light black instantly, eventually to brownish after 3 h. Then, to remove AgCl cores, the solution was washed by NH_4_OH for 3 times. The purified GYBs can disperse in water. The functionalization of GYB with amino group was achieved by using gold−sulfur bonding. 1 mg of dry GYB dispersed in 300 μl cysteamine hydrochloride dissolved in ethanol. The solvent evaporated in a vacuum oven and modified GYB redispersed in water, denoted as cys-GYB.

### Characterizations of GYBs

The average size and zeta potential of nanoparticles were analyzed by dynamic light scattering (DLS, Nano-ZS, Malvern). Samples were dispersed into water in glass cuvette. The size distribution of nanoparticles was measured by the light hit particles in a period. Field-emission scanning electron microscope (FE-SEM, JSM-7000F, Japan), a transmission electron microscope (TEM, JEM-2100, Japan) was applied to observe the morphologies of nanoparticles. The elemental mapping of nanoparticles was performed by the energy dispersive spectroscopy (EDS) of TEM. For SEM analysis, all the samples were dried on the silicon wafers at room temperature and gilded with an ultrathin platinum layer on the wafer to enhance the image quality taken in the experiments through the intensive electronic sputtering. For TEM analysis, nanoparticles were dried on the copper grid and took digital pictures of several locations on the grid to observe the lattice of crystallite and obtain a representative set of images. High-resolution X-ray photoelectron spectrometer (HRXPS, PHI Quantera SXM, Japan) can determine the surface composition of nanoparticles.

### Microfluidic chip manufacturing

The chip network was designed using geometric modeling software (AutoCAD, Autodesk Inc., Sausalito, CA, USA). We produce a cross-section pattern on the surface of the PMMA substrate (Kun Quan Engineering Plastics Co. Ltd., Taiwan) by utilizing a CO_2_ laser micromachining system (LES-10, Laser Life Co. Ltd., Taiwan). The microchip (6.5 cm (*L*) × 3.5 cm (*W*) × 0.4 cm (*H*)) consisted of two separated PMMA plates. Several through holes were drilled in the PMMA plates. The plate was washed by D.I. water with ultrasonic for 30 min and dried with N_2_ stream gas. The plates were pressed together by the elliot folder and bonded at 105 °C for 30 min. In the final, poly(etheretherketone) (PEEK) tubes were inserted into holes and firmed by glue.

### Synthesis of GelMA

Briefly, 5.0 g of type B bovine skin gelatin (Sigma-Aldrich, 10% wt/v) was dissolved in 50 mL of di-ionic water and mixed by a magnetic stirrer at 60 °C. 1.0, 2.5, and 5.0 mL of methacrylic anhydride was slowly added to gelatin solution for 3 h at 60 °C, respectively. The reaction was stopped by diluting the solution to a 5-fold volume. The solution was dialyzed in 12–14 kDa cutoff tubing at 40 °C twice a day for 5 days to remove unreacted methacrylic anhydride (MA) and salts. Lyophilization of solution for 4 days until gain a porous white foam and stored the foam at −20 °C.

### Generation of microbeads by microfluidic chip

The aqueous phase (7.5 wt% GelMA solution with 0.5 wt% Irgacure 2959 in D.I. water) and the oil phase (5wt% Span80 in paraffin oil) were prepared then centrifuged at 6800 × *g* to remove the impurities. The flow rates were well controlled by syringe pumps and the droplet formation were monitored by an inverted optical microscope. The microbeads were crosslinking by UV light (365 nm, Series 1500, OmniCure). These microbeads were washed with hexane three times and PBS three times to remove the oil and surfactant. The size of the microbeads was measured by NIH image J software. The microbeads were preserved at 4 °C until further used.

### Elastic modulus measurement

The samples of crosslinked GelMA at different concentration were prepared as previously described^32^. Then, the cylindrical hydrogels were compressed at a rate of 20% strain/min by a mechanical tester (Instron 5542). Elastic modulus was calculated as the slope of the linear region corresponding with 0-5% strain of a stress−strain curve.

### Preparation of conductive microporous hydrogel

To prepare the conductive microporous hydrogels (CMH), different volume ratios of microbeads and cys-GYBs solution (1 mg/ml) were added in plastic tubes and thoroughly mixed by vertexing. After mixing, the tubes were centrifuged for 1 min at 5800 × *g* and the colloidal gels were confined at the bottom of the tubes.

### Coverage of MBs analysis

To quantify the coverage of the GYBs on the sphere, we divided the GYBs positive area by the total area of the sphere surface. Each of the sphere surface area in the image was segmented manually using lasso tool in the Avizo 9.4 (ThermoFisher) and the GYBs positive area on top of each sphere in all the images were further segmented by a specific gray level range (3500-65535, 16 bit). To quantify the area of segmented mask for each sphere and the GYBs coverage area on top of it, we use Material Statistics module to quantify the area of each mask. It calculates the voxel numbers inside a labeled area, which can be transformed into area after multiplied by known voxel size in each image.

### Electrical conductivity and LED emitting test

To measure electrical conductivity of each gel, the cylindrical hydrogels were prepared (cross-sectional area: 3.14 × 1.0 cm^2^; length: 1.0 cm). Then, two-terminal electrical resistance (Ω) was monitored using digital multimeter (Dawson), and the electrical conductivity was calculated as follows: Electrical conductivity (S cm^−1^) = *L*/*R* × *A*, where *R* is (electrical resistance, Ω), *A* is (Atra of cross-sectional (cm^2^) = 3.14 × radius^2^), and *L* is (length, cm). Furthermore, LED emission was visualized while hydrogel was serially connected.

### In vitro cell culture

The N_2_A and astrocyte cells were maintained in DMEM containing with 10% fetal bovine serum (FBS) and 1% penicillin at 37 °C and 5% CO_2_ incubator. The culture medium was replaced every three days with fresh one. For sub-culturing cells, the spent DMEM were suctioned from 10 cm dish. The cells were washed with warm PBS gently and warm 1.0% trypsin-EDTA were added into dish for 3 min. The number of cells were calculated by using cell counter and trypan blue. The NSC cells were isolated from ED 14−15 Wistar rat embryos using a previously described protocol with modification^49^. The NSC cells were incubated in serum-free Dulbecco’s modified eagle’s medium (DMEM)-F12 and N_2_ supplement (100 mg/mL of human apotransferrin, 25 mg/mL of insulin, 30 nM of sodium selenite, and 20 nM of progesterone at pH 7.2).

### In vitro experiments

To examine the cellular uptake of cys-GYBs, and the influence of HFMF, we utilized QD to label the cys-GYBs. The N_2_A, astrocyte and NSC cells were incubated on the glass cover slips in the wells for 24 h. Then, we added 2 mL medium including 50 μL of vehicles after the cultivation on the glass cover slips. Subsequently, we incubated the cells at 37 °C, and then, 5% CO_2_ was filled in the incubator and keeping for different times. When we accomplished the requirement we designed, we would remove the medium in the wells and wash with PBS twice. Subsequently, the cells were fixed with 4% formaldehyde, and then, immersed in 0.1% of Triton X-100 in PBS solution for 30 min. Eventually, we prepared F-actin (300 units/mL) and DAPI (1 μg/mL) to stain the cellular actin cytoskeleton and nuclei for 1 h, respectively. After finishing all the above steps, we mounted the sample on glass slide and observed with fluorescence microscopy. The progress of observing the cellular uptake of cys-GYBs-HFMF was as same as the above method, and for the group which was irradiated by HFMF would be irradiate HFMF after added the cys-GYBs for 4 h.

### Cell viability assay

Cytotoxicity of N_2_A, astrocyte cells were treated at the different concentration of cys-GYBs. We incubated cells in 96 well microplates with 200 μL of medium and each well cultivated 1 × 10^4^ cells for 24 h. Then, we replaced the old medium with different concentration of cys-GYBs and new medium into each well. We cultured the cells for the next 24 h. Eventually, we added 10 μL of prestoblue into each well and incubated the cells for 1 h. The absorbance was detected with a microplate reader at a wavelength 570 nm. The data was contrasted with the untreated one. For NSC group, we used a trypan blue exclusion assay with a hemocytometer. NSCs were cultured in the medium with 20 ng/mL of basic fibroblast growth factor (bFGF) and treated at the different concentrations of cys-GYBs. To investigate the survival of NSCs at the different concentrations of cys-GYBs, live/dead staining was used to visually evaluate the survival status. The same process as described. After incubation for 48 h in 24-well plates, the culture medium was replaced with 200 μL serum-free medium containing 0.5 μM of calcein AM and 3 μM of propidium iodide (PI), respectively. After incubating for 15 min at 37 °C, and examined under a laser scanning confocal microscope (ZEISS LSM-780) and calcuated by 5 z-axil images of one NSC spheroid.

### Immunocytochemistry of differentiation

NSC cells were cultured for 7 days (w/o 100 ug/ml cys-GYBs and HFMF) in vitro. Cells were fixed in ice-cold 4% paraformaldehyde in PBS for 20 min and washed three times with PBS. After fixation, the following antibodies were diluted in PBS containing 10% Fetal bovine serum (FBS) and 0.5% Triton X-100. In brief, the cells were stained for 2 h at 37 °C with the primary antibody solutions. The obtained cells were stained as follows: (1) MAP-2: mouse anti-microtubule-associated protein 2 (1:1000 dilution; Chemicon), (2) GFAP: rabbit anti-glial fibrillary acidic protein (1:1000 dilution; Chemicon). After washed by PBS three times, The secondary antibodies were as follows: (1) rhodamine-conjugated goat anti-mouse IgG (1:250 dilution; Chemicon). (2) FITC-conjugated donkey anti-rabbit IgG (1:250 dilution; Chemicon). The secondary antibody was stained for 2 h by using and then washed three times with PBS. Next, stained the cells with DAPI for 10 min and then washed three times with PBS. The morphology of all the stained cells was observed using a laser scanning confocal microscope (ZEISS LSM-780). The intensity and the alignment of regenerating axons at each segment (proximal, middle and distal) were analyzed by image J software.

### Analysis of differentiation percentage of neural cells

To calculate the percentage of differentiated cells of each phenotype, the intensity of glial fibrillary acid protein (GFAP) and microtubule-associated protein 2 (MAP2)-positive cells were calculated in the area out of the neurospheres to determine the astrocyte and neuron percentages in each field, respectively. The images of all the stained cells were observed using a laser scanning confocal microscope (ZEISS LSM-780).

### Quantification of sprouts in the NSC spheroid

After 48 h incubation, cells sprout from NSC spheroids was identified as DAPI. Take fluoresce images from the sprouts of at least ten randomly selected spheroids per test condition. The images can be analyzed using a suitable program such as J image analysis software. This allows an area of interest to be drawn around the outgrowths, and the sprouts. are then highlighted by the software. The number of sprouts and the average for each treatment are then compared to the untreated control.

### In vivo experiment

The surgical procedure was performed in accordance with the protocol approved by the Animal Care and Use Committee, National Tsing Hua University, Hsinchu, Taiwan. The GelMA MBs were immersed in PBS for full swelling. C57BL/6 mice (Female, 7 weeks) were divided into four groups (*n* = 6): (1) phosphate buffered saline (PBS), (2) MBs, (3) CMH, and (4) CMH + HFMF groups. An electric drill was first used to drill a hole on the skull when applying TBI to mice. Afterwards, a punch with 2 mm in diameter was adopted to give a 1.5 mm injury in depth. After removing the brain tissue, 10 μL of MBs or CMH (based on fractional void volume of CMH, the volume of gel was 61%(v/v) of gel in PBS solution) were injected by syringe. For HFMF treatment group, HFMF was applied 5 min/day until mice were sacrificed. To quantify the results, 25 slices per animals and 3 ROIs in lesion/trauma regions were randomly chosen and calculated.

### Brain collection and immunofluorescence staining

At 7, 40, and 82 days postsurgery, the harvested brain tissue was fixed overnight in 4% paraformaldehyde in 0.1 M PBS at pH 7.4 for 12 h then transferred into in 30% sucrose solution for 2 days for dehydration. The brain embedded in optimum cutting temperature compound (OCT; Surgipath FSC22, USA) at −80 °C overnight. The samples were sliced as horizontal cryostat sections (15 μm in thickness) and then stained for immunohistochemical analysis. In brief, the frozen sections were incubated at room temperature to melt frozen section compound for 30 min. Then Immersed slides in the methanol at 4 °C for 10 min and washed by PBS three times to remove OCT. Then, the sections were stained overnight at 4 °C with the primary antibody solutions. The primary antibody was as follows: (1) NF200 (1:200, rabbit IgG1, Abcam) for regenerated neuron filament. (2) GFAP (1:200, rabbit polyclonal, Abcam) for astrocyte cells. (3) IBa1 (1:200, goat polyclonal, Abcam) for microglia cells. (4) CD31 (1:200, mouse polyclonal, Abcam) for vascular cells. The secondary antibody was stained with Alexa 488 (1:200, goat anti-mouse IgG1) and Alexa 488 (1:200, goat anti-rabbit IgG1) for 2 h and then washed with PBS three times. Finally, stained the sample with DAPI for 10 min and then washed three times with PBS. The morphology of all the stained sections was observed using a laser scanning confocal microscope (ZEISS LSM-780).

### BOLD-fMRI acquisition and data analysis

For meaningful comparison with their corresponding neurological outcome with different CMH implants treated acute TBI model, functional recovery of brain regions in and around focal injuries was examined with the evoked Blood-oxygenation-level-dependent (BOLD) fMRI activity in response to a non-nociceptive stimulus^50, 51^. Nevertheless, anesthesia drug- and dose-dependently modulated the neural activity triggering changes in BOLD signal intensity. Many studies suggested the dexmedetomidine plus low-dose isoflurane anesthesia was conducted on the rodent to result in the boxcar-like BOLD response with consistent hemodynamic patterns in the contralateral sensorimotor cortical regions induced by the somatosensory stimulation^51–53^. In this study, the mice were anesthetized with 0.03 mg/kg dexmedetomidine hydrochloride subcutaneously after induction with 3% isoflurane (Attane, Minrad Inc., Bethlehem, PA, USA) mixed with 20% O_2_, 75% N_2_, and 5% CO_2_ for 5 min for sedation under anesthesia. Following appropriate sedation, the animal was transferred into the magnet and secured in a customized holder for MRI scan with maintenance at 0.3% of isoflurane. Isoflurane was turned off after 30 min of the dexmedetomidine hydrochloride injection, keeping the mixed air for breathing during fMRI scanning. To prevent the body temperature reduction caused by anesthesia, a circulating pad was used to maintain warmth at 36.5–37.5 °C. Animal respiration and heart rate should be maintained within 130–150 and 300–400 rpm, respectively, in this study.

For forepaw electrical stimulation, two 30 G needle electrodes were inserted into the web space of digits 4/5 of the forepaw. A biphasic electrical current with a pulse width of 0.2 ms was delivered and applied by using an isolated stimulator (S48, Grass Technologies, West Warwick, RI, USA) with an isolated constant-current unit (PSIU6, Grass Technologies, West Warwick, RI, USA). The intensity and frequency of the electrical stimulation were 1 mA and 12 Hz, respectively, as commonly used to evoke the contralateral sensorimotor cortical BOLD activation in dexmedetomidine sedated rodents^53, 54^.

MRI was performed by a 7-T scanner with a 30-cm diameter bore (Bruker Biospec 70/30 USR, Bruker Corp., Ettlingen, Germany), and a linear volume coil was used to transmit the radio frequency pulses. To receive the radio frequency signal, a planar surface coil (T7399V3, Bruker Corp., Billerica, MA, USA) was placed directly over the head. For anatomical scan, a rapid acquisition with refocused echoes (RARE) T2 images were acquired (TR = 2,500 ms, TE = 33 ms, matrix size = 256 × 256, field of view (FOV) = 20 × 20 mm^2^, 14 coronal slices, thickness = 0.5 mm, number of excitations = 4) to uniform slice positioning. To minimize the BOLD signal loss around the injury region prior to fMRI scan, the magnetic field homogeneity was optimized through the standard localized shimming with first-order shims on an isotropic voxel of 7 × 7 × 7 mm^3^ encompassing the imaging slices^55^. Following achieving shimming procedure to optimize the local BOLD sensitivity over cortical regions, fMRI images were acquired by a gradient echo- planar imaging (GE-EPI) sequence (TR = 2,000 ms, TE = 20 ms, FOV = 20 × 20 mm^2^, matrix size = 80 × 80, bandwidth = 200 kHz, 14 coronal slices, and thickness = 0.5 mm).

For acquiring BOLD responses, a standard stimulus paradigm consisted of a boxcar design starting with a resting period of 20 s (baseline), followed by five cycles of a 20-s stimulus OFF period and a 20-s stimulus ON period each, in which each session contained 120 GE-EPI images, including 5 stimuli blocks and 6 control blocks without forepaw stimulation. The GE-EPI images contained 10 dummy scans, followed by a total of 110 volumes.

fMRI data preprocessing was performed by the FMRIB Software Library v5.0 (FSL 5.0; http://www.fmrib.ox.ac.uk/fsl) and the Analysis of Functional NeuroImages (AFNI) software (http://afni.nimh.nih.gov/afni). All fMRI data were reconstructed into 4D data images (3D volume images and time series) and following the steps below. AFNI’s 3dAutomask algorithm was first used to strip mouse skull to exclude the influences of non-brain voxels. Following removing dura, skull and various non-brain structures from EPI images, the slice-timing correction was used to realign to the first scan of GE-EPI images by the AFNI’s 3dVolreg^56^ with default iterated least-square minimization^57^, which could reduce the influence of brain motion within the scanner. The coregistration of RARE T2 images and GE-EPI images was performed using a 6-parameter (rigid body) spatial transformation. fMRI data sets were normalized by using 12-parameter affine transformations. The spatial smoothing was performed using a full-width half maximum (FWHM) Gaussian kernel of 0.4 mm. Linear detrending was applied to correct the signal drift.

The BOLD activation maps were created using the 3dDeconvolve general linear model function with the given boxcar design of the stimulation paradigm by AFNI^56^. The normalized BOLD responses were determined by the evoked BOLD signals over the injured regions of interests (ROIs) on the left hemisphere then dividing the BOLD signals over the equivalent non-injured (contralateral) ROIs on the right hemisphere. In this study, ROIs were selected based on the basis of clusters of functional activations of primary somatosensory cortex of forelimb (S1FL) and primary motor cortex (M1) according to the Allen mouse brain atlas^58^. The activation map of the BOLD fMRI responses to forepaw stimulation was determined by Z-test analysis^59^. The significant level was set at Z > 2.3 (*p* < 0.05), and the group-level BOLD fMRI Z‐maps was then superimposed on the RARE T2 anatomical images of Allen mouse brain atlas^58^.

In this study, our stability of fMRI measurements for BOLD signal time courses and localization of the activation could be found as long as the above experimental methods including the anesthetic protocol with optimal dose ranges and imaging time points, parameter manipulation for somatosensory stimulation, and fMRI preprocessing and analysis were followed^60^, which therefore resulting in the reproducibility and spatial agreement of BOLD responses to contralateral forepaw stimulation on S1FL and M1 cortical regions over different mice.

Ensuring that our fMRI-BOLD experiment with enrolling the enough number of animals used in each group to ensure reproducibility and spatial agreement of stimulus-evoked BOLD responses was a critical aspect of experimental design. Power, or the ability to reliably detect magnitude differences between evoked BOLD responses between stimulus ON and OFF, was used to determine their corresponding effect sizes and powers using open source toolbox, G^∗^Power (version 3, Institut fürExperimentelle Psychologie, Dusseldorf, Germany)^61^, and Cohen’s *d* equation^62^. The magnitude of evoked BOLD signal in response to the forepaw stimulation showed a significant difference between stimulus ON and OFF in the ROIs of M1 (PBS: 1.7 ± 0.2%, *p* < 0.05, *n* = 6; MBs: 1.9 ± 0.2%, *p* < 0.05, *n* = 6; CMH: 1.5 ± 0.1%, *p* < 0.05, *n* = 6; CMH + HFMF: 2.2 ± 0.3%, *p* < 0.05, *n* = 6) and S1FL (PBS: 4.1 ± 0.2%, *p* < 0.05, *n* = 6; MBs: 4.3 ± 0.3%, *p* < 0.05, *n* = 6; CMH: 4.0 ± 0.1%, *p* < 0.05, *n* = 6; CMH + HFMF: 5.8 ± 0.4%, *p* < 0.05, *n* = 6), the power were more than 0.8 in M1 (PBS: effect size = 4.966, power = 0.986; MBs: effect size = 9.118, power = 0.959; CMH: effect size = 6.591, power = 0.999; CMH + HFMF: effect size = 12.329, power = 0.997) and S1FL (PBS: effect size = 5.784, power = 0.998; MBs: effect size = 11.931, power = 0.995; CMH: effect size = 8.541, power = 0.999; CMH + HFMF: effect size = 14.907, power = 0.999). According to the power analyses and effect size test, there were sufficient statistical powers (> 0.8) and medium to large effect sizes each group as shown in Supplementary Table 1, which complied with the Three Rs (3Rs) principle for more ethical use of animals in this study^63^.

### Western blotting

After the second MRI scan, all implanted animals were sacrificed through decapitation. Their brains were harvested from the skull to investigating the protein secreted in trauma brain areas. The brain tissues with lesion were dissected from the left brain of the groups of PBS, MPs, CMH and CMH + HFMF group. Protein samples were extracted in ice-cold lysis buffer (50 mM of Tris-HCl, pH = 7.5, 0.3 M of sucrose, 5 mM of EDTA, 2 mM of sodium pyrophosphate, 1 mM of sodium orthovanadate, 1 mM of PMSF, 20 μg/ml of leupeptin, and 4 μg/ml of aprotinin) and then separated through SDS-PAGE (30 μg), and trans-blotted onto polyvinylidene difluoride membranes (Millipore, Billerica, MA, USA). The membranes were hybridized with the primary antibodies as fellows: (1) β-actin: rabbit IgG1 (1: 5000), (2) NeuN: rabbit IgG1 (1:1000), (3) BDNF: rabbit IgG1 (1:1000). Then, the blots were washed and incubated with HRP-conjugated goat anti-rabbit IgG antibody (1:1000 dilution), and developed by Luminata Forte Western HRP substrate (Millipore, Billerica, MA, USA). The images were acquired using the luminescence imaging system (LAS-4000, Fujifilm, Tokyo, Japan). A gel analysis plug-in for the Image J software. In this study, the protein levels were normalized to the internal control of β-actin. The protein weight of β-actin was 42 kDa; pro-BDNF and mature-BDN were 35 kDa and 17 kDa, respectively; NeuN was 46 and 48 kDa.

### Animal behavior test

C57BL/6 mice (Female, 7 week) were divided into four groups (n = 6): (1) PBS, (2) MBs, (3) CMH, and (4) CMH + HFMF groups. The cylinder test followed the approaches in previous study^64^. In brief, the forelimb asymmetry was measured by observing the movements of mice in 3-min intervals in a transparent, 18-cm wide, 30-cm-high transparent cylinder. The cylinder was large enough to offer movement and small enough to enhance the rearing and wall exploration. Furthermore, a mirror on the bottom of cylinder was placed to observe and record forelimb moves while the mice faced away from the detectors. After rearing and wall exploration, a landing was scored for the first limb to contact the ground or for both limbs once the mice exhibited a simultaneous touch. The unimpaired and impaired limb was calculated for scoring. The ratios of use of the impaired limb were subtracted from percentage use of the unimpaired limb to estimate the limb bias score. Moreover, wall exploration and landing movements were also evaluated. Animals were tested at 4, 14, 21, 28, 49, and 82 days after TBI following implantation.

The grid test was carried out in a 2.5 × 2.5 cm^2^ square wire fence with a grid area of 12 cm × 36 cm × 10 cm (length/width/height) was used. During the experiment, mice were placed on the fence alone and the number of foot faults, when the foot was not supported and went through the grid hole, was calculated by number of foot faults/(foot faults + number of non-foot-fault steps) × 100. Animals were tested at 4, 14, 21, 28, 49, and 82 days after TBI following transplantation.

### Statistics and reproducibility

Statistical analyses were performed by GraphPad Prism (GraphPad Software, version 8.0) on data from three or more independent experiments. Error bars indicate SD for three or more independent experiments. The differences between groups were analyzed by one-way ANOVA followed by Dunnett’s or Tukey’s multiple-comparison test as indicated in figure legends. *P  <  0.05, **P  <  0.01, ***P  <  0.001, ****P  <  0.0001 were considered statistically significant. The full statistical results are provided in the Source data file. All experiments in Fig. 1c; 2b-c; 2l; 4c; 8b and Supplementary Figures 1a; 2a-b; 3a; 5a-f; 7; 8; 10; 11a-c; 13b, 14 were repeated at least three times independently with similar results.

### Reporting summary

Further information on research design is available in the Nature Research Reporting Summary linked to this article.

## Supplementary information

Supplementary Information

Reporting Summary

## Data Availability

The authors declare that the data supporting the findings of this study are available within the article, source data, and its Supplementary Information. Other relevant data are available from the corresponding author upon reasonable request. A reporting summary for this article is available as a Supplementary Information file. Source data are provided with this paper.

## Source data

Source Data
