# Supplementary material for: Wireless charging-mediated angiogenesis and nerve repair by adaptable microporous hydrogels from conductive building blocks
Source: Nat Commun. 2022 Sep 2;13:5172. doi: 10.1038/s41467-022-32912-x (PMC9440098; doi:10.1038/s41467-022-32912-x)
Supplement: Supplementary file 1 — Supplementary Information [file 41467_2022_32912_MOESM1_ESM.pdf]

Supplementary Information for

Wireless Charging-Mediated Angiogenesis and Nerve Repair by Adaptable Microporous Hydrogels from Conductive Building Blocks

Ru-Siou Hsu[1], Ssu-Ju Li[2], Jen-Hung Fang[1], I-Chi Lee[1], Li-An Chu[1,3], Yu-Chun Lo[4], Yu-Jen Lu[5,6,*], You-Yin Chen[2,4,*], Shang-Hsiu Hu[1,*]

[1] Department of Biomedical Engineering and Environmental Sciences, National Tsing Hua University, Hsinchu 300044, Taiwan
[2] Department of Biomedical Engineering, National Yang Ming Chiao Tung University, Taipei 112304, Taiwan
[3] Brain Research Center, National Tsing Hua University, Hsinchu 300044, Taiwan
[4] The Ph.D. Program for Neural Regenerative Medicine, College of Medical Science and Technology, Taipei Medical University, Taipei 11031, Taiwan
[5] Department of Neurosurgery, Chang Gung Memorial Hospital, College of Medicine Chang Gung University, Taoyuan 33305, Taiwan
[6] College of Medicine, Chang Gung University, Kwei-San, Taoyuan 33302, Taiwan

Correspondence should be addressed to Yu-Jen Lu, (email: alexlu0416@gmail.com), You-Yin Chen (email: irradiance@so-net.net.tw) and Shang-Hsiu Hu (email: shhu@mx.nthu.edu.tw).

Keywords: Conductive hydrogel, Adaptable, Porous scaffold, Neuron modulation, Nerve repair

Supplementary Table 1. The effect sizes and powers for BOLD signal changes in M1 and S1FL cortical regions in four groups (n = 6).

Brain region	***Group***	***Effect size***	***Power***
**M1**	PBS	4.966	0.986
	MBs	9.118	0.959
	CMH	6.591	0.999
	CMH+HFMF	12.329	0.997
**S1FL**	PBS	5.784	0.998
	MBs	11.931	0.995
	CMH	8.541	0.999
	CMH+HFMF	14.907	0.999

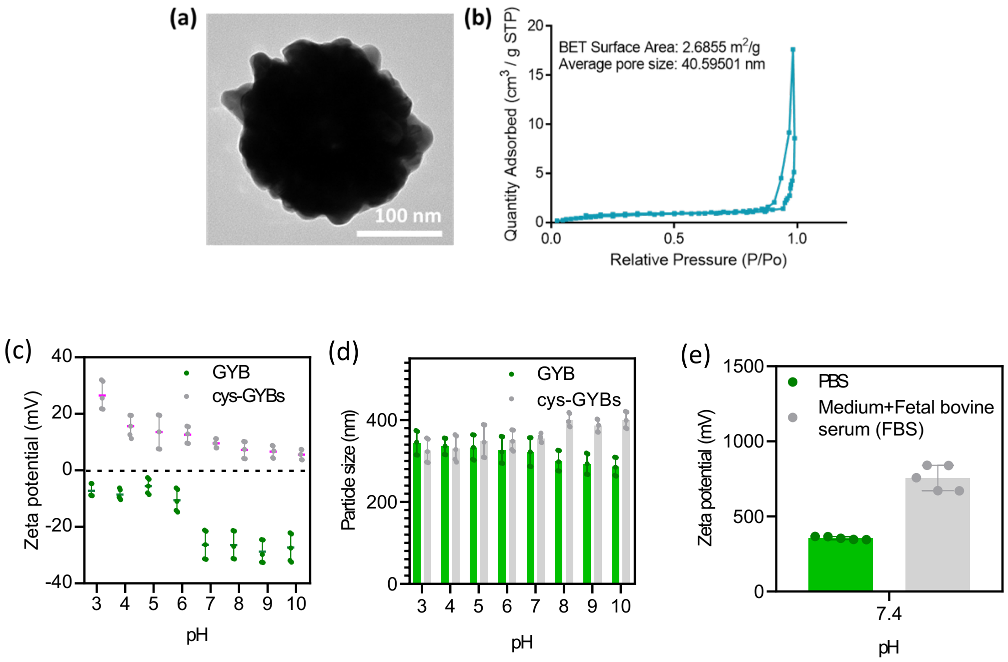

Supplementary Fig. 1. (a) Transmission electron microscopy (TEM) image and (b) Brunauer–Emmett–Teller (BET) analysis via nitrogen absorption/desorption isotherms of GYBs. (c) Zeta potentials and (d) sizes of GYBs and cys-GYB at various pH. Error bars represent mean ± s.d., n = 5. (e) The sizes of cys-GYB in the presence of medium and 10% Fetal bovine serum (FBS). Error bars represent mean ± s.d., n = 5.

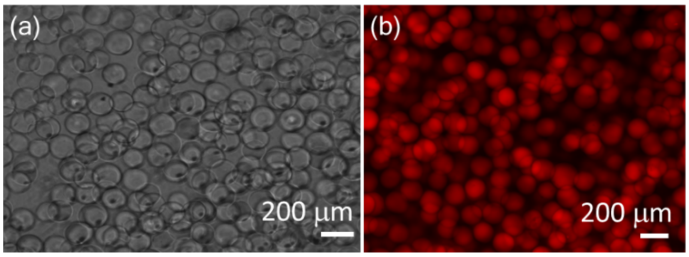

Supplementary Fig. 2. (a) 7.5 wt% GelMA MBs in PBS photoed by optical microscope. (b) 7.5 wt% GelMA MBs stained by RITC in PBS phototed by fluorescence microscope.

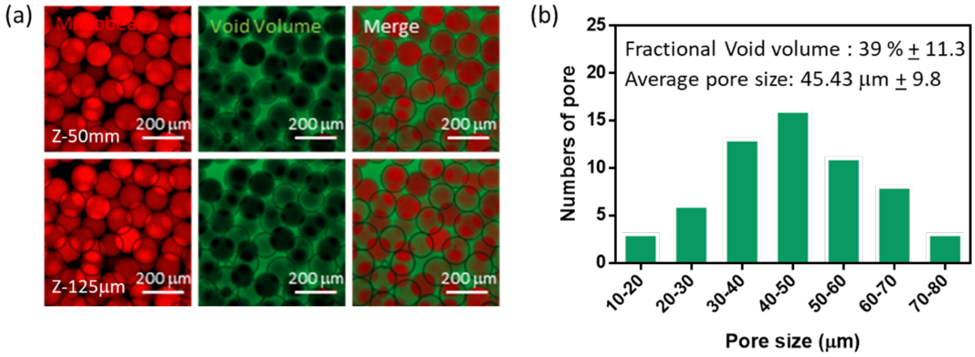

Supplementary Fig. 3. (a) The fluorescence image of CMH (red) with fluorescence solution (green). The scale bar in fluorescence image is 200 μm. (b) The distribution of number with pore size.

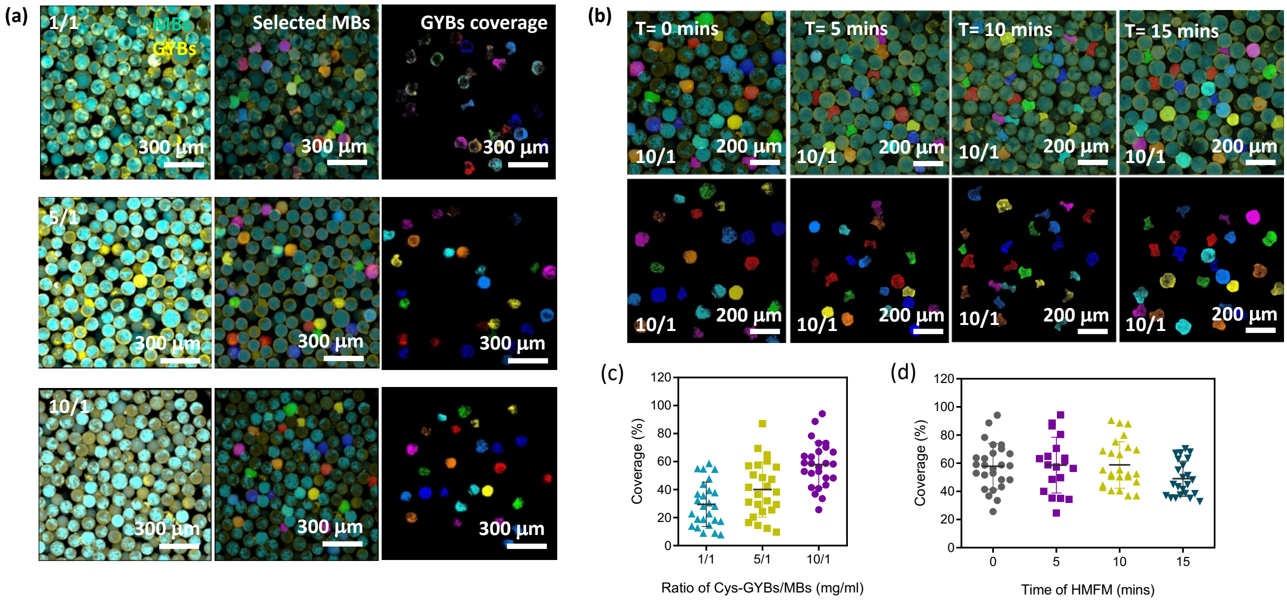

Supplementary Fig. 4. (a) The quantification of fluorescence of cys-GYBs fractional coverage on selected MBs. (b) The quantification of fluorescence of CMH under HFMF at different time periods. The fractional coverage of CMH (c) at different ratios of cys-GYBs and MBs and (d) treated by HFMF for various time. Error bars represent mean ± s.d., n = 20.

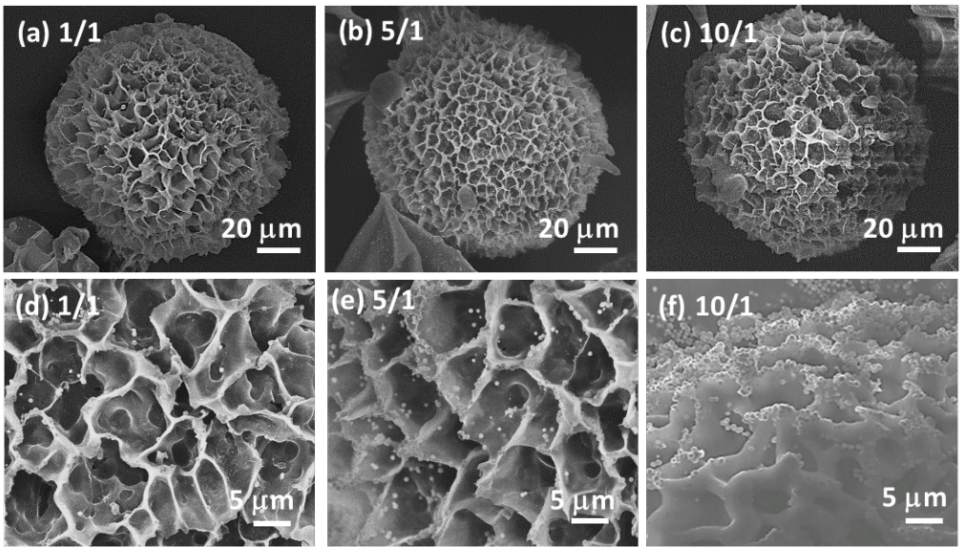

Supplementary Fig. 5. The SEM images of CMH at (a,d) 1/1, (b,e) 5/1 and (c,f) 10/1 mg/ml of GYBs to MBs.

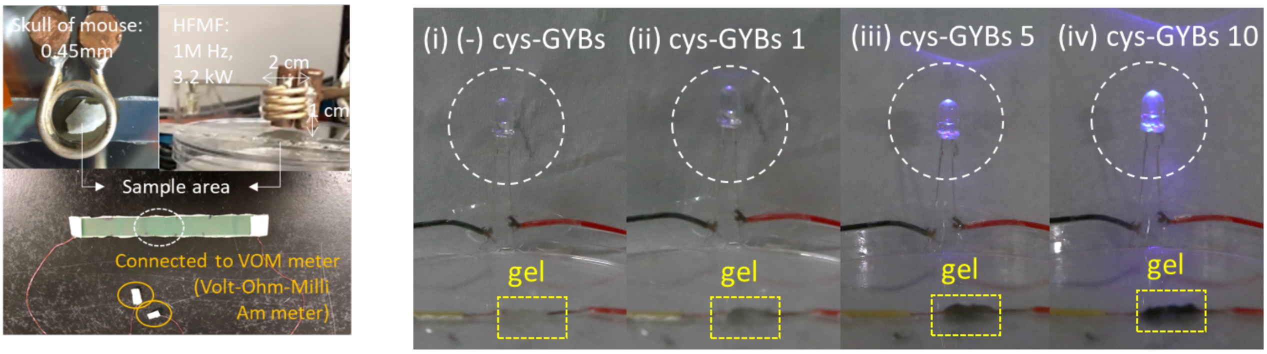

Supplementary Fig. 6. (Left panel) The measurement setup of induced current upon a HFMF treatment. To avoid the influence of wires-induced currents, the 10 cm of FTO plate was used as the subtract, and the wires were connected in the non-current-induced area. (Right panel) LED emitting tests in the electrical circuit serially connected to the various hydrogels.

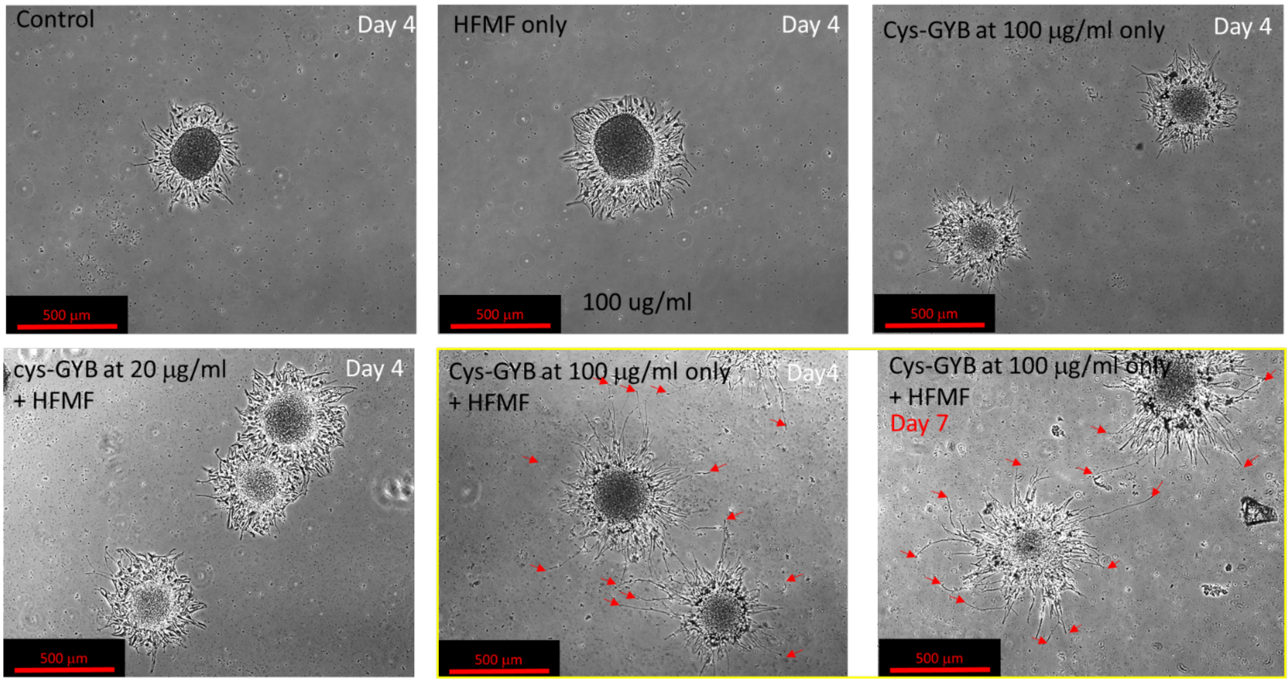

Supplementary Fig. 7. Optical images of isolated mouse neural stem cells treated by cys-GYBs at day 4 and day with a treatment of HFMF.

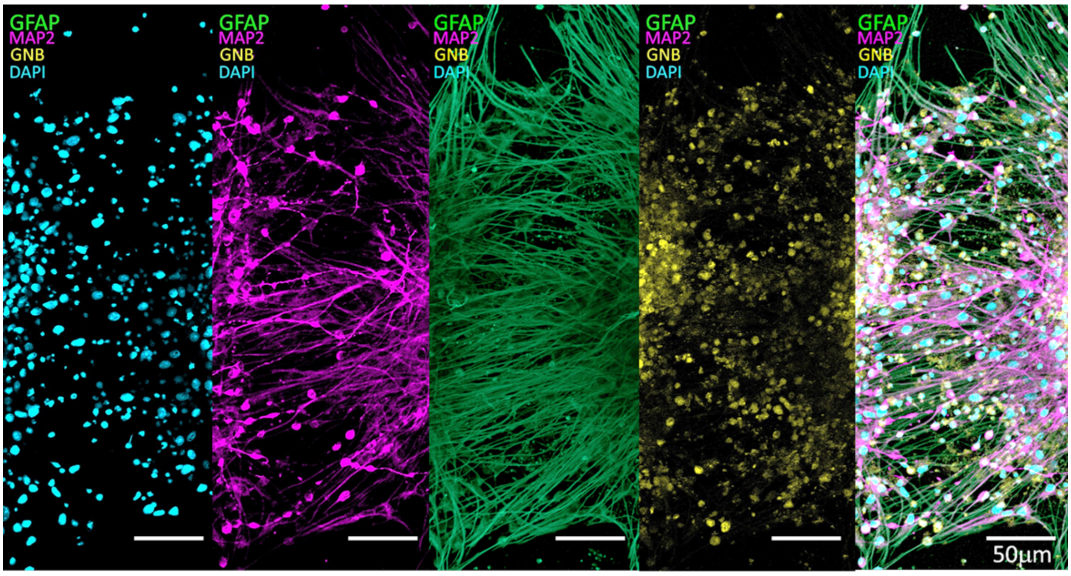

Supplementary Fig. 8. Magnification of CLSM images of cys-GYBs+HFMF group.

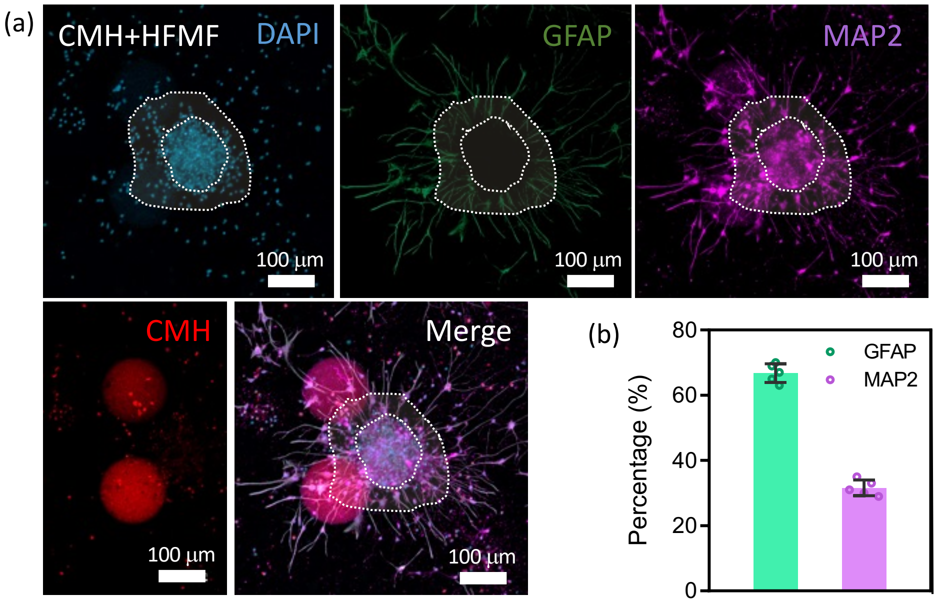

Supplementary Fig. 9. (a) CLSM images of of CMH-treated NSCs upon a 5 min of HFMF treatment per day for 7 days. (b) Quantification of the percentage of differentiation into neurons and astrocytes from NSCs under various treatments for 7 days. Error bars represent mean ± s.d., n = 5.

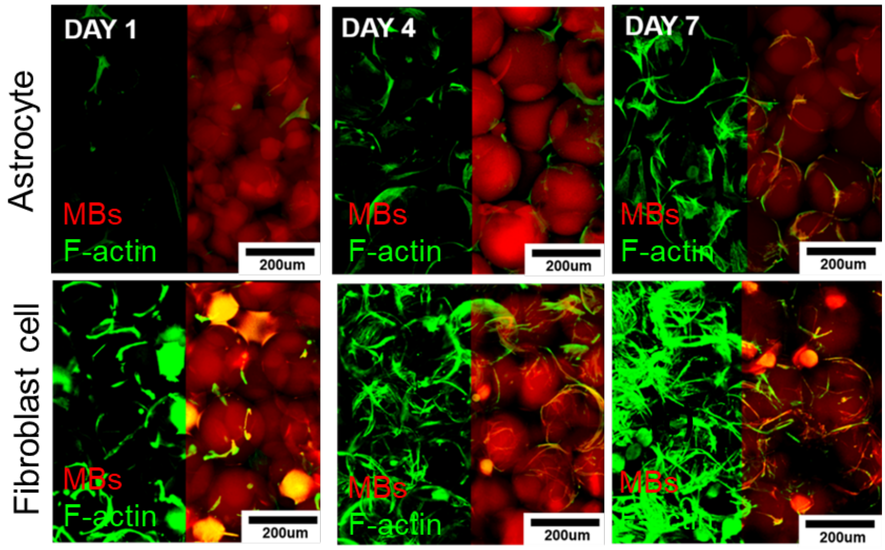

Supplementary Fig. 10. Fluorescence images demonstrating the formation of 3D cellular networks during seven days of culture *in vitro*.

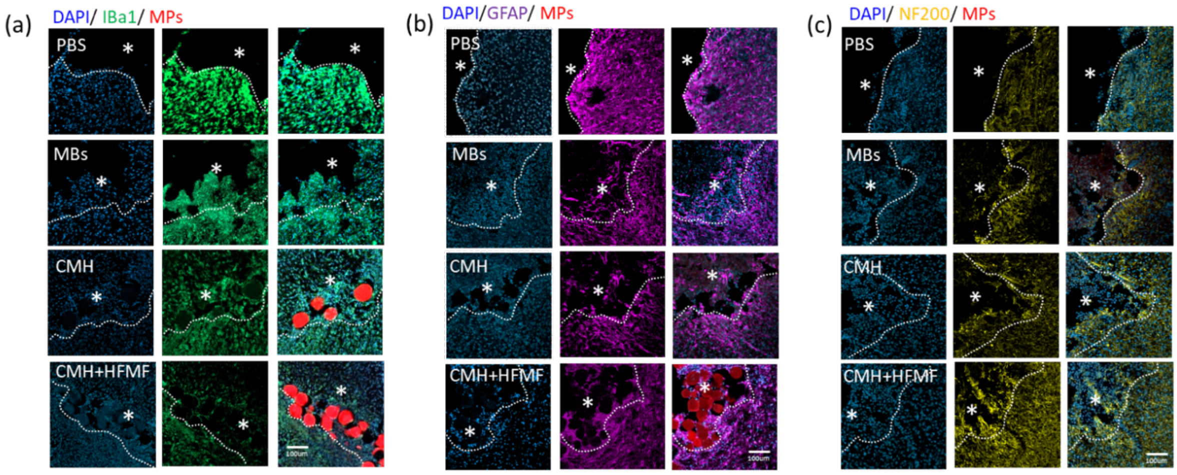

Supplementary Fig. 11. Trauma boundaries were defined by the intact and fluorescence intensity of the tissues. In the brain tissue, the distribution of nuclei was more uniform than trauma tissues. Furthermore, GFAP-expressed trauma tissue performed stronger than surrounding tissues. (a) Fluorescence images of Iba-1, (b) GFAP and (c) NF200 immunostaining after treatment with PBS, MB, CMH and CMH+HFMF).

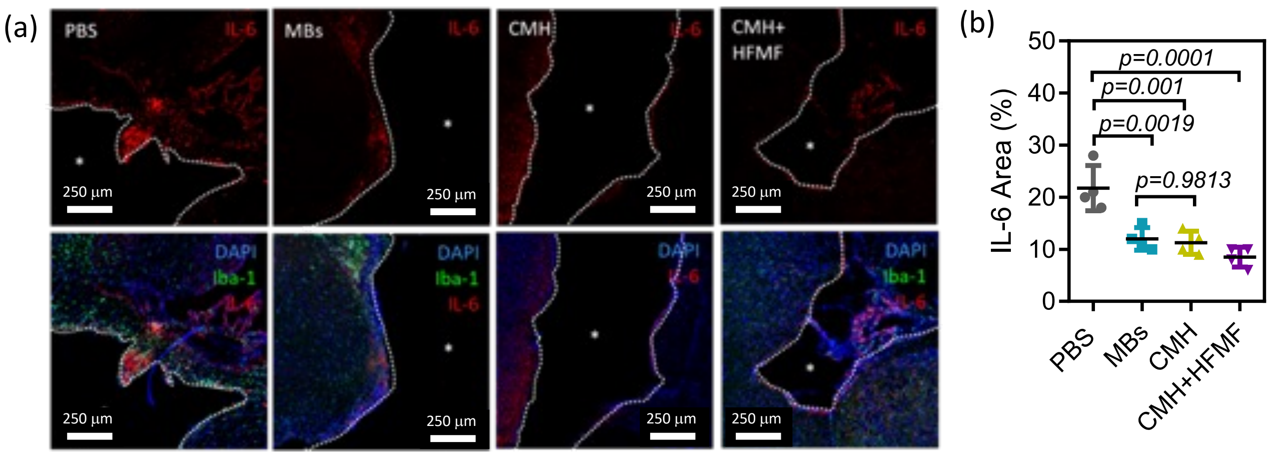

Supplementary Fig. 12. (a) CLSM images and of the expression of interleukin-6 (IL-6) of the peri-trauma area treated by PBS, MBs, CMH and CMH+HFMF at 7 days postsurgery. (b) Quantification of the expression of interleukin-6 (IL-6). Error bars represent mean ± s.d., n = 5, one-way ANOVA with Tukey's multiple comparison test.

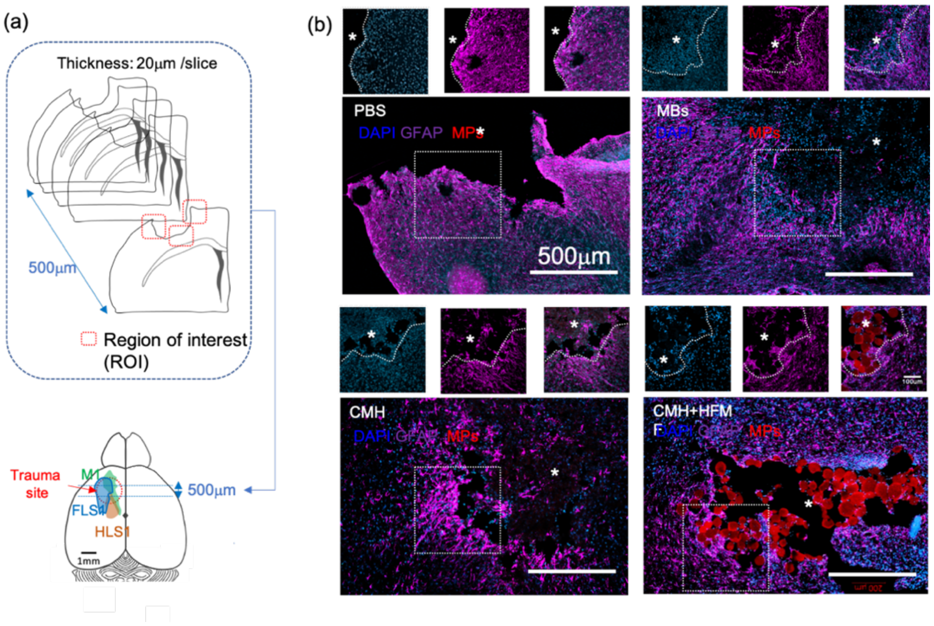

Supplementary Fig. 13. Analysis of the GFAP positive response in terms of scar thickness, astrocytic infiltration in the infarct area, and the positive area for GFAP signal in both infarct and peri-infarct regions. (a) Scheme for image analysis of ROI and (b) GFAP stained in vivo for astrocyte studies.

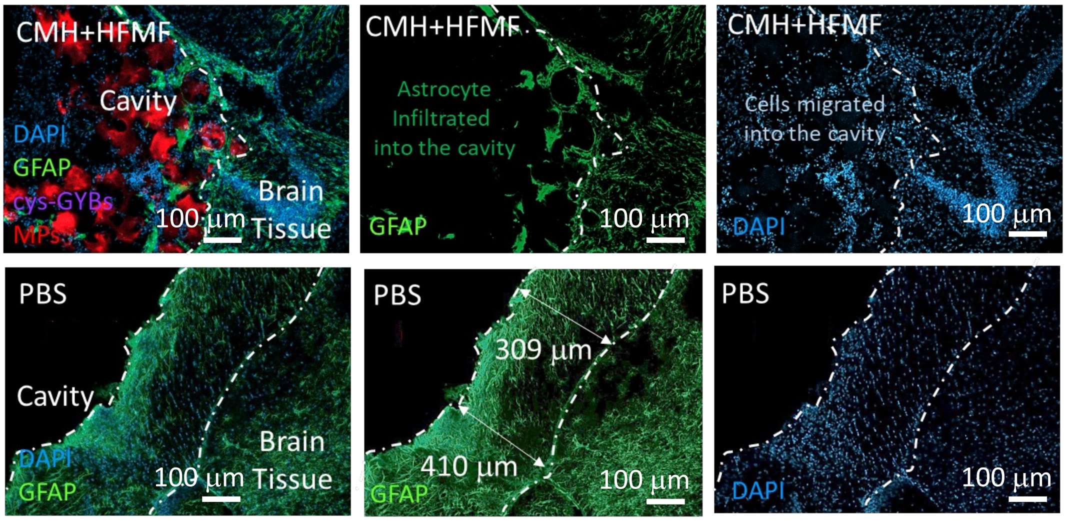

Supplementary Fig. 14. Astrocyte cells infiltrated and ingrowthed into the cavity after 30 days of implantation in brain. Fluorescent images of astrocyte (GFAP) in green, nucleic (DAPI) in blue and MPs (RITC) in red around the trauma site at 30 days postsurgery. The upper panel shows less scar formation and astrocyte infiltration in the CMH+HFMF-treated group. The lower panel of control group shows obviously scar around the cavity.

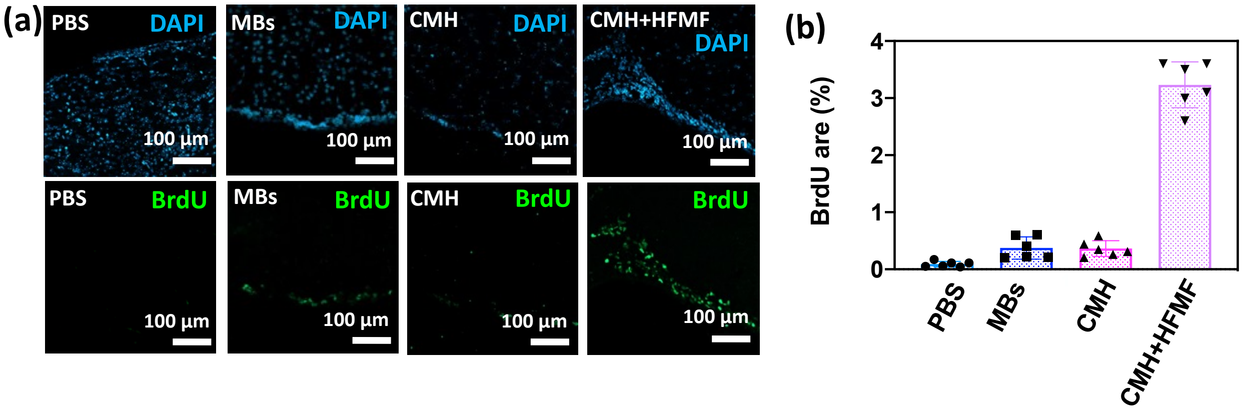

Supplementary Fig. 15. (a) Fluorescence images of BrdU immunostaining after treatment with PBS, MB, CMH and CMH+HFMF at 30 days postimplantation. (b) The quantification results of fluorescence images of BrdU signals (n = 6, mean ± s.d.).

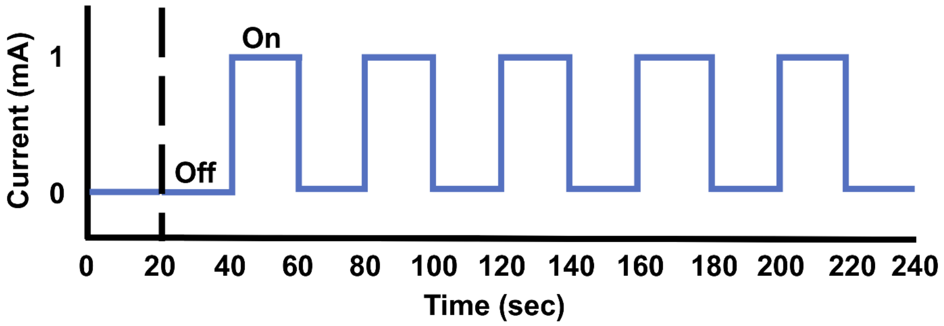

Supplementary Fig. 16. Stimulation paradigm of BOLD-fMRI. The stimulation paradigm consisted of five electrical stimulation blocks interlaced with six blocks without electrical stimulation. Given resting duration of 20 sec and stimulus duration of 20 sec. The electrical stimulation current of 1 mA with a plus width of 25 μs at 12 Hz.

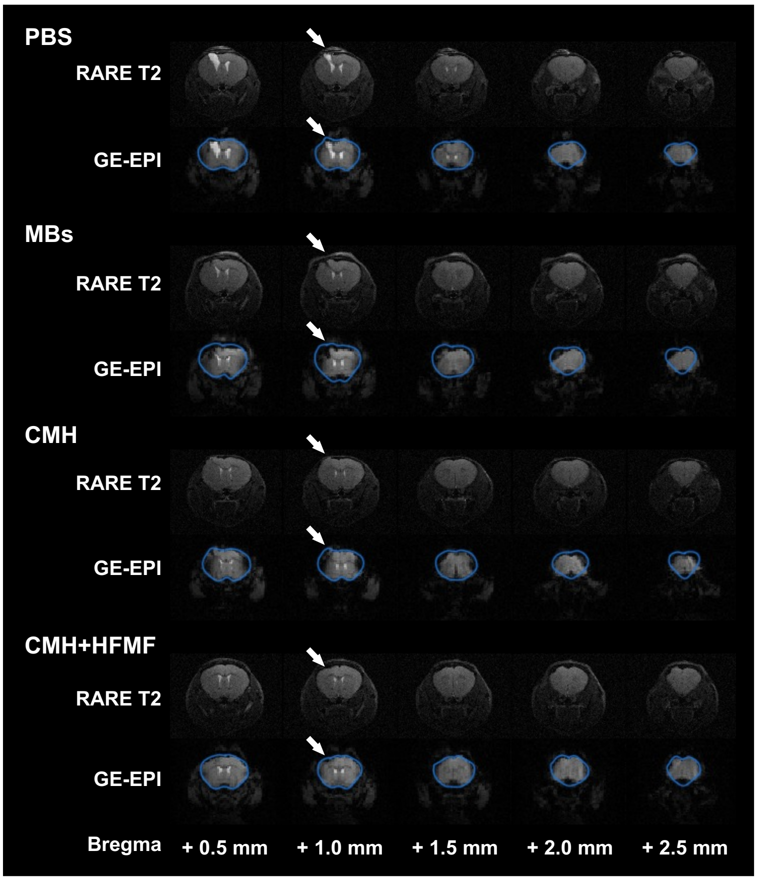

Supplementary Fig. 17. RARE T2 and GE-EPI raw images without skull stripping of PBS, MBs, CMH and CMH+HFMF, respectively. The trauma sites were highlighted using white arrows in raw images of GE-EPI and T2-RARE in the same mouse each group. The EPI images were aligned more closely with their corresponding brain masks of RARE T2 anatomic reference (blue line) in the CMH+HFMF treated mouse. However, EPI image distortions near the trauma site (coronal images from bregma +1.0 mm to bregma +2.5 mm) were not fully recovered in the groups of PBS, MBs and CMH, appearing few image voxels or signal loss inside the brain mask.
